# Multicentre, multi-arm, double-blind randomised placebo-controlled dose-finding trial investigating the safety and Efficacy of MirococePt (APT070) In Reducing delayed graft function In the Kidney ALlograft (EMPIRIKAL-2): study protocol for a randomised controlled trial

**DOI:** 10.1136/bmjopen-2024-097029

**Published:** 2025-03-06

**Authors:** Theodoros Kassimatis, Alima Rahman, Fayyad Jaradat, Abdel Douiri, Marc Delord, Roseanna Greenlaw, Joanne Palmer Joyce, Jonathon Olsburgh, Muhammad Khurram, Abbas Ghazanfar, Simon Knight, Peter Friend, Sapna Shah, Hannah Killbride, Richard Smith, Steven Sacks

**Affiliations:** 1 Peter Gorer Department of Immunobiology, School of Immunology and Microbial Sciences, King’s College London, London, UK; 2 King’s Kidney Care, King’s College Hospital, London, UK; 3 Transplant, Renal and Urology Directorate, Guy's and St Thomas’ NHS Foundation Trust, London, UK; 4 Clinical Trial Management Service, Research and Development, Guy's and St Thomas’ NHS Foundation Trust, London, UK; 5 School of Life Course and Population Sciences, King’s College London, London, UK; 6 Department of Nephrology and Transplantation, The Royal London Hospital, London, UK; 7 William Harvey Research Institute, Queen Mary University of London, London, UK; 8 Renal and Transplant Unit, St George’s Hospital, London, UK; 9 Nuffield Department of Surgical Sciences, University of Oxford, Oxford, UK; 10 Kent Kidney Care Centre, East Kent Hospitals University NHS Foundation Trust, Canterbury, Kent, UK; 11 School of Immunology and Microbial Sciences, King’s College London, London, UK

**Keywords:** Renal transplantation, NEPHROLOGY, Randomised Controlled Trial

## Abstract

**Background:**

Up to 50% of kidney transplant patients are diagnosed with delayed graft function (DGF) following transplantation—the majority being linked to ischaemia reperfusion injury (IRI). DGF is traditionally defined as the requirement for dialysis during the first week after transplantation and is associated with inferior graft and patient outcomes. Local synthesis of complement components, largely by the renal tubule, plays a critical role in IRI. We have developed Mirococept, a membrane-targeted complement inhibitor, that can be administered to the donor kidney ex vivo prior to transplantation. After administration, Mirococept is retained in the donor organ, thereby minimising the risk of systemic side effects. We previously launched the EMPIRIKAL study aiming to evaluate the efficacy of Mirococept in reducing DGF in deceased-donor kidney transplantation (KT). The funding body recommended termination of the study to allow a dose-saturating study before the next stage of clinical evaluation. This was carried out in a porcine kidney model and led to a revised dosing regimen for EMPIRIKAL-2 (60–180 mg compared with 5–25 mg in the initial trial). The EMPIRIKAL-2 trial (REC 24/NE/0071) aims to identify the most safe and efficacious dose of Mirococept to reduce DGF rate in deceased-donor KT.

**Methods and analysis:**

EMPIRIKAL-2 is a Phase IIa multicentre double-blind randomised controlled trial (RCT) with an initial safety run. Participants will be recruited from renal departments at National Health Service tertiary hospital sites in the UK. The purpose of the safety run is to assess the tolerance of each of the three proposed Mirococept doses (60, 120 or 180 mg), before the RCT begins. Three patients will be assigned to each treatment dose, starting from the lower dose. The safety run will be considered successful if at least one dose can be taken forward to the RCT for comparison to placebo.

If safety is met, 144 participants (36 per arm excluding drop-outs) will be randomised to all doses meeting the safety criteria or placebo on a 1:1:1:1 basis. The primary endpoint is DGF, defined as the requirement for dialysis during the first week after transplantation. Safety evaluation will include the monitoring of laboratory data and the recording of all adverse events. Immunosuppression therapy, antibiotic and antiviral prophylaxis will be administered as per local centre protocols. Enrolment in the RCT is anticipated to take approximately 12 months, and patients will be followed-up for 12 months.

**Ethics and dissemination:**

The study has been approved by the Northeast - Newcastle and North Tyneside 2 Research Ethics Service Committee, REC reference 24/NE/0071. The results of the study will be reported and disseminated at international conferences and in peer-reviewed scientific journals. Once published, a lay summary of the results will be made available to participants who request this information.

**Trial registration number:**

ISRCTN14279222. Registered on 4 July 2024.

**Protocol version:**

2.0 dated 9 May 2024.

**STRENGTHS AND LIMITATIONS OF THIS STUDY**

- This is a multicentre, multi-arm, double-blind randomised placebo-controlled dose-finding trial with an initial safety run.
- The initial safety run will establish the best tolerated dose of Mirococept which will be taken forward to the randomised controlled trial for comparison to placebo.
- Mirococept, given by ex vivo administration, is retained in the donor kidney, thus minimising the risk of systemic side effects.
- The study is powered to detect a reduction of delayed graft function from 50% down to 30%, which may not apply to other centres where there is a different case mix.

## Background

### DGF in kidney transplant patients

 Up to 50% of kidney transplant patients are diagnosed with delayed graft function (DGF) following transplantation—the majority being linked to ischaemia reperfusion injury (IRI). DGF has traditionally been defined as the requirement for dialysis within the first week after transplantation, although over 10 definitions have been recorded in the literature.^1^ The incidence of DGF has risen substantially over the last decade due to a marked increase in the use of donation after circulatory death (DCD) and expanded criteria donor (ECD) kidneys.^2 3^ DGF is associated with higher rates of acute rejection, reduced kidney transplant survival and prolonged hospitalisation.^4 5^ The relative risk of graft loss and death at 5 years in patients with DGF is 1.89 and 1.64, respectively.^6^ Strategies to prevent IRI and consequent DGF are therefore more relevant today than 10 years ago.

### Role of complement in IRI

The complement system plays a critical role in all forms of solid organ IRI.^7^ The reperfusion of the ischaemic kidney induces a proinflammatory reaction in which the complement system activation plays a central role.^8^ Complement-depleted or deficient animals exhibit reduced post-ischaemic acute renal failure and chronic nephropathy.^9^,^11^ The evidence strongly suggests that local synthesis of complement components largely by the renal tubule has a prominent role in activating local inflammatory processes and mediating graft immunogenicity, more so than the circulating pool produced by hepatic synthesis.^9^ The central step in complement activation is the cleavage of C3 to C3b by C3 convertase. Membrane-bound C3b binds to activated factor B and forms the C3 convertase enzyme resulting in further C3 cleavage to C3b. This ultimately leads to C5a and C5b-9-mediated injury of the renal tubule, and C3a and C5a-mediated enhancement of the recipient immune response.^12 13^ In vivo, the aberrant complement activation is prevented by the regulators of complement activation (RCAs). RCAs are poorly expressed on the tubule surface both in normal and ischaemic kidneys, making the tubule vulnerable to complement attack.

Together, these mechanisms provide a rationale for local therapeutic manipulation of C3 function during early transplantation in order to improve the short and possibly longer-term outcomes of renal grafts. Such a targeted approach would provide graft protection while avoiding the off-target effects of systemic complement inhibition in immunosuppressed recipients.

### Regulators of complement activation and therapeutic potential

Mirococept (APT070) is a highly effective complement inhibitor which is derived from CR1 (CD35). It consists of three functional units: the terminal three short consensus repeat domains of CR1 which contain its biological activities, a membrane associating peptide and a membrane-inserting myristoyl group.^14^ The last two units permit the binding to and insertion into the cell membrane. Mirococept is unique in that it is engineered to bind to cell surfaces in bulk^15^ and can block the complement system at the C3 level. It does not inhibit proteases generally, and its action is restricted to the complement system.

Mirococept’s selectivity derives from the extensive contact surface between the complement control protein domains of the CR1 fragment and C3b, which is a component of the alternative and classical pathway-derived C3 and C5 convertases. Mirococept inhibits these convertases by two highly specific mechanisms, one by inducing dissociation of the subunits of the convertases and the other by acting as a cofactor for the proteolytic breakdown of C3b by the endogenous serum protease factor I.

### Preclinical studies

Mirococept has undergone proof-of-concept evaluation in animal models of inflammation including rheumatoid arthritis,^16^ Guillain-Barré syndrome,^17^ intestinal ischaemia^18^ and myocardial ischaemia.^19^ We previously developed a strategy to administer Mirococept in the donor kidney, thereby avoiding any undesirable consequences from systemic complement inhibition.^10^ After being administered to the kidney ex vivo via the renal artery, Mirococept localises to the graft endothelial and epithelial cells. In a rat transplant model with 30 min of cold ischaemia, we showed that local inhibition of complement activation within the graft by Mirococept reduced complement-mediated injury and lowered the ability of the kidney to initiate acute cell-mediated rejection.^10^ Specifically, Mirococept reduced inflammatory injury and allograft rejection, thus improving graft function in the short term (throughout the first week) and long term (20 weeks). With prolonged cold ischaemia (16 hours), treatment led to reduced complement deposition and inflammation in transplanted kidney, with graft losses reduced to 36.4%, compared with 73.3% in control-treated organs. Moreover, the treated rat kidneys that remained viable after transplantation had a more rapid rate of recovery than controls, with 33% relative reduction in area under the curve (AUC) of creatinine over the first 7 days.^20^

### Clinical studies

A summary of the clinical studies of Mirococept can be found in table 1.

**Table 1:** Summary of clinical studies of Mirococept to date

Study	Cohort	Dose	Outcomes
Phase I	42 healthy human volunteers	2–100 mg intravenous	Good tolerability at doses up to 100 mg
Phase IIa pilot	16 kidney transplant patients	10 mg	Trend towards faster recovery in IGF group
Phase II (EMPIRIKAL)	First cohort of 80 kidney transplant patients	10 mg	Trend towards faster recovery in IGF group

IGFimmediate graft function

A Phase I study in human volunteers treated with incremental doses (2–100 mg) of intravenous Mirococept showed a favourable safety profile (see Safety below).

A Phase IIa pilot study in 16 kidney transplant patients confirmed the feasibility of the ex vivo administration of Mirococept at 10 mg/kidney prior to transplantation and showed a trend towards more rapid recovery of graft function compared with control organs.^21^

The first EMPIRIKAL Phase II study set out to evaluate the dose and efficacy of Mirococept in reducing DGF rates in deceased donor kidney transplantation.^22^ The design was to test a dose range of 5–25 mg in cohorts of 80 patients for each dose with a maximum number of seven cohorts. This dose range was scaled up from data in a rat transplant model in which renal functional recovery was the principal measure of efficacy.^10 20^ The results with the first dose (10 mg, infused into the donor organ), however, showed no effect on DGF rate compared with the control group (although there was an encouraging trend towards faster recovery of renal function associated with Mirococept in patients with immediate graft function (IGF). The funding body—the Medical Research Council, UK—recommended termination of the study to allow a dose-saturating study before the next stage of clinical evaluation.

### Dose-finding pig study

We therefore conducted a porcine kidney study, using a higher dose range to determine kidney localisation and safety in terms of local toxicity and washout of unbound reagent at the higher loading dose.^23^ Peak binding of fluorescently labelled (PEG-FAM)-Mirococept to the renal tubules occurred after intra-arterial administration of 80 mg Mirococept, compared with 20, 40 and 160 mg.

Allowing for the average weight of human kidney, the dose-equivalent of Mirococept (80 mg) used in the pig study is 120 mg for human kidney,^24^ which is 12-fold higher than the 10 mg dose used in Cohort 1 of EMPIRIKAL. The study also showed no histological or functional evidence of toxicity after a 3-hour normothermic machine perfusion (NMP) and no evidence of drug release in excess of the dose tolerated by healthy volunteers (see Safety below).

## Safety

The safety of Mirococept has been demonstrated in preclinical studies as well as in Phase I and II clinical trials.

A Phase I, randomised, double-blind, placebo-controlled, dose escalation study to evaluate the safety, tolerability, pharmacokinetics and pharmacodynamics of Mirococept in healthy men was carried out according to Good Clinical Practice (GCP). Mirococept was administered as a single intravenous infusion. There were seven dose levels (2, 5, 10, 20, 40, 70 and 100 mg) and six participants in each dose level. Four participants received active drug and two received placebo. There were no deaths or serious adverse events (AEs) and the number of treatment-related AEs was low up until the 100 mg Mirococept dose was administered, where there was increased reporting of events in three out of four participants receiving the active medication. For this dose level, the most common issues were general disorders (fatigue, headache) and nausea with one occurrence of fever. The lack of any consistent pattern in the AEs for doses lower than 100 mg Mirococept indicated good tolerability. There were no treatment-related changes in vital signs and clinical laboratory parameters. In particular, there was no evidence of oedema or swelling at any dose level in contrast to the findings in rats.

The Phase IIa pilot and the EMPIRIKAL studies (see section on Clinical studies above) confirmed the feasibility and safety of ex vivo administration of Mirococept, as validated in preclinical models.^21 23^ The dose used for the pilot and the EMPIRIKAL studies was chosen on the basis of animal data.^10 20^ To our knowledge, the EMPIRIKAL study was the first study to evaluate the efficacy of such a procedure for a complement inhibitor in preventing DGF in deceased-donor kidney transplantation. Neither Mirococept nor Mirococept-specific IgG were detected in the sera from transplant recipients in Cohort 1 of the trial, and the small, transient drop in serum complement activity observed after surgery was similar in both treated and placebo groups and is likely to be the result of the surgical procedure and not a result of Mirococept in the circulation.

The porcine kidney model identified the dose of Mirococept (80 mg) to achieve optimal binding for a pig kidney weighing approximately 150 g. This dose had no observable detrimental effect on the histological integrity of the organ (acute tubular injury), both before and after 3 hours of NMP.^23^ Importantly, measurements of blood flow and intrarenal resistance in the isolated perfused pig kidney also showed no adverse effects related to Mirococept, supporting the histological interpretation.^23^ Moreover, our washout studies in the porcine kidney model showed that only a small fraction (about 12%) of the loading dose of Mirococept is released ex vivo. This is in agreement with past studies on isolated porcine kidney carried out by Ad Protech—the biotechnology company (1997–2004) where under Richard Smith’s direction Mirococept was invented. The estimated release for the human loading dose of 180 mg would therefore be 23 mg of Mirococept (12.5% of 180 mg), which is well within the range considered safe from the Phase I trial with up to 100 mg given intravenously to healthy volunteers.

The optimal Mirococept dose for a pig kidney (weighing approximately 150 g) was 80 mg. Scaling up for the average weight of human kidney, the equivalent dose of Mirococept is approximately 120 mg, which is 12-fold higher than the 10 mg dose used in Cohort 1 of the clinical study.

### EMPIRIKAL-2

Taken together, the EMPIRIKAL Cohort 1 data and subsequent pig kidney data support the need for definitive clinical evaluation of the efficacy of Mirococept in human kidney transplantation. EMPIRIKAL-2 aims to determine the optimal dose (60, 120 or 180 mg, deduced from pig data^24^) of Mirococept to reduce DGF in adult patients undergoing deceased-donor renal transplantation compared with placebo controls so that this can be taken forward to a pivotal Phase III study. A more accurate estimate of effect size, currently estimated conservatively at 20%, will also allow correct powering of the Phase III pivotal study. The trial will also underpin the rationale for analysis of the long-term clinical benefits.

## Patient public involvement

Kidney transplant patients were involved in reviewing and providing feedback on the patient information sheets (PIS), patient invitation letter (PIL) and informed consent form (ICF). The study protocol and PIS were also presented at a departmental transplant, renal and urology research board meeting at Guy’s and St Thomas’ NHS Foundation Trust. This board includes patient representatives and meets regularly to review and provide feedback on the design and feasibility of studies.

The Trial Steering Committee (TSC) will include a patient representative. The TSC will meet every 6 months and will have oversight of the conduct of the trial from set-up to closure. Having a patient representative on this committee will ensure that the conduct of the trial is mindful of patients’ views and feelings towards this research.

## Methods/design

This is a multicentre, multi-arm double-blind randomised placebo-controlled dose-finding trial with an initial single-centre safety run. Participants registered on the kidney transplant list and considered eligible based on pre-screening by the clinical care team will be recruited from renal departments at National Health Service (NHS) tertiary hospital sites in the UK. It has an operationally seamless adaptive design to evaluate the safety and superiority of Mirococept in reducing DGF in deceased donor kidney transplantation as compared with placebo.

### Safety run

The purpose of the single-centre safety run is to assess the tolerance of the study drug at three different dose levels and confirm which doses should be taken forward to the randomised controlled arm of the trial.

Nine participants will be enrolled into the safety run at Guy’s Hospital, assigned to one of the three treatment doses (60, 120 or 180 mg) and followed-up for 12 months. Participants one to three will receive kidneys treated with 60 mg of Mirococept; participants four to six will receive kidneys treated with 120 mg; and participants seven to nine will receive kidneys treated with 180 mg. Recruitment within each dosing group will be staggered so that the interval between participant dosing will be a minimum of 48 hours and up to 2 weeks, to allow a full safety assessment for each participant. In between the dosing groups, there will be a review of safety data before dose escalation to the next level. The aim is to have at least three participants in each dosing group who complete the safety run, so drop-outs will be replaced. We anticipate the estimated duration of the safety run recruitment period to run for 4 months in total. To eliminate selection bias, kidney offers for this part of the study will be considered as they come.

The data-safety review in between each dosing group of the safety run will focus on the following outcomes:

1. Non-perfusion of the donor kidney (day 0) (or identified on routine Doppler ultrasound, day 1) in the absence of any technical failure or hyperacute rejection.
2. DGF lasting for more than 2 weeks despite adequate perfusion.

By way of explanation, non-perfusion is rare, affecting 1–2% of all kidney transplants. DGF, however, can affect up to 50% of grafts, of which 75% become dialysis independent within 2 weeks.^25^

A formal review by the Data Monitoring Committee (DMC) will be triggered in the following circumstances:

1. More than one of three occurrences of non-perfusion (day 0/day 1) in each dosing group, or two occurrences in a row between groups, during the safety run, absent of technical failure or hyperacute rejection.
2. Two or more patients per group with DGF lasting for more than 2 weeks.

For each dose cohort, measurement of serum Mirococept at specific time points over the first 4 days post-transplant will indicate the amount of the drug (if any) entering the recipient’s systemic circulation relative to the tolerated range as defined by the Phase I trial (where healthy volunteers received Mirococept in escalating doses).

Measurement of serum complement activity (CH50) will detect changes in complement in the same period. Evidence of reduced systemic complement activity (expected to be minimal and transient) would not itself be a safety issue because doses up to 100 mg Mirococept given intravenously were well tolerated in the Phase I study. Moreover, the relative safety of near-complete inhibition of CH50 by anti-C5 antibody is well documented (eg, in patients with haemolytic-uraemic syndrome). However, recording of Mirococept and CH50 levels will form part of the safety report.

After completion of the safety run, a DMC meeting will be convened to review the safety data before progression to the RCT is permitted. It will be considered successful if the DMC considers at least one dose can be taken forward for comparison to placebo. This will provide a go/no-go gate to the main recruitment of the multicentre RCT.

### Multi-arm randomised placebo-controlled trial

If all three doses are found to be safe and well tolerated in the safety run, 144 participants (36 per arm) will be randomised to receive a kidney perfused with Mirococept or placebo on a 1:1:1:1 basis, stratified by centre and donor type. 90 participants (30 per arm) will be required if two of the proposed doses are well-tolerated, and 40 participants (20 per arm) will be randomised if only one dose can be taken forward for comparison with placebo.

The participants enrolled in the safety run will not be included in the RCT analysis.

The primary outcome of DGF will be assessed at seven completed days post-transplantation, and participants will be followed-up for 12 months. The design will be a double-blind, randomised placebo-controlled trial and will therefore minimise bias.

Safety assessments during this randomisation stage will include routine renal function and urine tests, with further investigation (eg, renal ultrasound and Doppler) as appropriate and complement activity and serum Mirococept levels as per trial protocol.

In the event that DGF rate and safety outcomes are similar in more than one dosing group, the final decision on which dose to take forward to the next clinical trial will include the secondary outcome measures. Of these, the most important are: functional DGF rate (where the fall in creatinine is less than 10% per day for three consecutive days); and AUC (area under the curve) based on the drop-in serum creatinine level in the first 2 weeks after transplantation where the participant does not require postoperative dialysis. If the combined primary and secondary endpoints of efficacy and the safety outcomes do not discriminate between the two doses, then the lower of the two doses would be taken forward to the next trial.

If this trial is successful and subject to funding being secured, the best dose will be taken forward into a definitive two-arm trial against placebo. The experimental unit, follow-up time points, randomisation and blinding will be the same in this second stage of the adaptive design.

## Trial objectives and endpoints

### Primary objective

To identify the most safe and efficacious dose of Mirococept to reduce DGF rate in deceased-donor renal transplantation so this can be taken forward in a pivotal Phase III study.

### Secondary objectives

1. To evaluate the efficacy of Mirococept to improve the rate of recovery in grafts with DGF or IGF independent of dialysis.
2. To determine if treatment influences renal function at 12 months (a surrogate of long-term graft outcome) and incidence of acute rejection episodes during this time.
3. To estimate more accurately the effect size, currently estimated conservatively at 20%. This will also allow correct powering of the Phase III pivotal study.

### Primary endpoint

1. DGF—requirement for dialysis during week 1 post-transplantation.

### Secondary endpoints

1. Functional DGF—failure of serum creatinine to decrease by at least 10% daily on three consecutive days in week 1 post-transplantation (excludes patients with biopsy-proven rejection or calcineurin inhibitor (CNI) toxicity in week 1 post-transplantation).
2. Number of dialysis sessions during the first 30 days post-transplantation.
3. Duration of DGF.
4. Mean calculated GFR (glomerular filtration rate) (Chronic Kidney Disease Epidemiology Collaboration) at 12 months post-transplantation.
5. Mean calculated creatinine clearance (Cockcroft-Gault) at 12 months post-transplantation.
6. Primary non-function—permanent lack of graft function for 3 months post-transplantation.
7. AUC of serum creatinine on days 1–14 post-transplantation.
8. Biopsy-proven acute rejection at 6 and 12 months post-transplantation.
9. Biopsy-proven CNI toxicity within 12 months post-transplantation.
10. Duration of post-transplantation hospital stay.
11. Number and duration of any hospital admissions during the first 12 months post-transplantation.
12. 1-year graft survival (censored and uncensored for death with functioning allograft).
13. 1-year patient survival.

## Preparation of the kidney and administration of Mirococept

The following will take place in the operating theatre and will be undertaken by delegated study personnel:

- Using 1000 mL of the cold storage solution, the first 500 mL will be used to flush the organ prior to administration of the IMP (investigational medicinal product). Currently, two cold storage solutions are available on the market: University of Wisconsin and histidine-tryptophan-ketoglutarate solution. We have tested the stability of Mirococept in both solutions, and it was found to be equally stable in both (data from these stability studies provided in the Investigator’s Brochure (IB). During the trial, only one cold storage solution will be used across all sites for every participant recruited to the study.
- The kidney will be placed in a container of ice slush for bench work.
  The vials of IMP take approximately 15 min to thaw, so should be removed from the dedicated −20°C freezer and thawed by standing at room temperature and then manually swirled initially, again after a further 10 min and finally checked visually. After this time, the solutions should be clear or slightly opalescent.
- While the kidney is in ice slush, all six vials of the IMP kit should be drawn up from the participant’s allocated drug kit and added into the remaining 500 mL of the cold storage solution (500 mL should be left in the 1000 mL bag following the initial 500 mL flush).
- Used vials should be discarded in the theatre sharps bin and destroyed as per local standard operating procedures (SOPs). The empty kit box should be retained for accountability checking by the clinical research associate.
- Vial box tear-off label should be removed and affixed to the printed randomisation email for reconciliation purposes.
- The Mirococept or placebo perfusion bag should be affixed with the appropriate label.
- Once the IMP/placebo is mixed with the cold storage solution, the fluid is then perfused through the kidney via the renal artery(s), under 1 m hydrostatic pressure.
- The perfusion will be conducted via a cannula held in place manually such that as much perfusion solution as possible drains through the kidney.
- If there is more than one renal artery, and they are of sufficient size, the IMP should be distributed equally through each of the arteries according to their estimated proportion of the kidney that they vascularise.
- The administration of IMP should take approximately 15 min.
- Following perfusion, the cannula is removed and the kidney repacked for cold storage.
- No post perfusion flush or arterial or renal vein clamping will be required.
- The transplantation procedure will continue in the standard manner.

## Concomitant medication

For management of concomitant therapies, please refer to the IB. No adverse effects including potential interactions are anticipated. A complete listing of all concomitant medication received during the trial will be recorded in the relevant electronic case report form (eCRF).

Participants in this trial will receive the local standard treatment for those undergoing a renal transplant. This encompasses pre-surgery medication, general anaesthesia, recovery from anaesthesia, pain control medication and anti-infective therapy (viral, fungal and bacterial). Immunosuppression therapy should be given as per local standard protocol.

Anaesthesia-related medication will not be regarded as concomitant medication.

Other experimental drug therapies will not be allowed for the duration of the trial.

## Selection of participants, recruiting and consent

### Selection of participants

**Figure 1:**
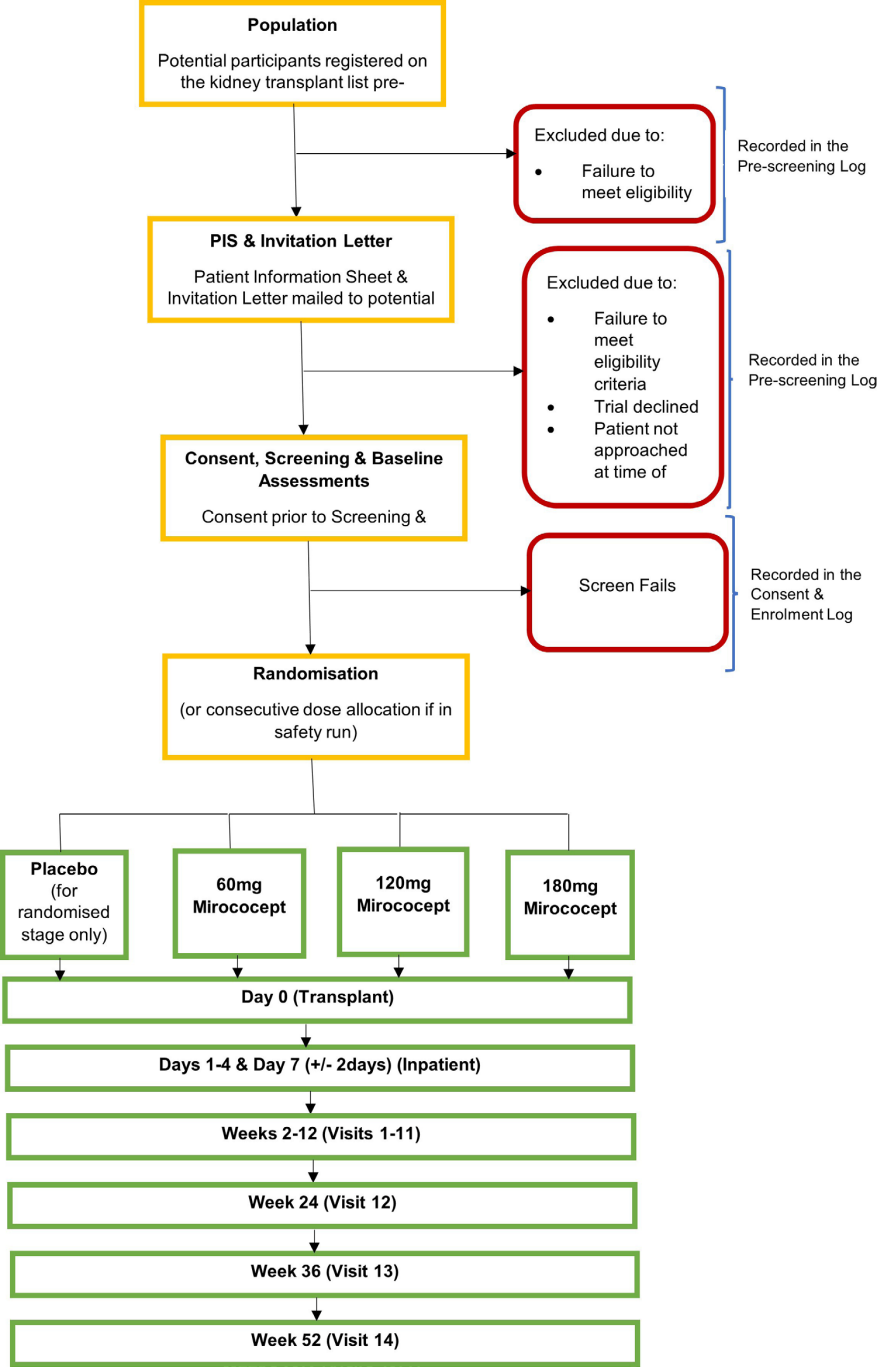
Trial flowchart. PIS, patient information sheets.

Participants will be recruited from renal departments at NHS tertiary hospital sites in the UK. The inclusion and exclusion criteria are shown in table 2. The trial flowchart is shown in figure 1. Potential participants registered on the national active kidney transplant waiting list will be identified by review of their medical records and initially approached by members of the direct care team. Due to the emergency and unscheduled nature of deceased donor transplantation, it is important for potential participants to have had the opportunity to consider the trial in advance. When a donor kidney becomes available, the time frame from notification to patient admission and transplantation is often on the same day. Therefore, potentially eligible participants from the kidney transplant waiting list will be posted or emailed a PIS along with a PIL ahead of any offer of a donor kidney. Contact details of the local site research team will be provided in the PIS giving an opportunity for potential participants to discuss the study in advance and have any questions answered.

**Table 2:** Inclusion and exclusion criteria

Inclusion criteria	Male or female patients aged ≥16 years or over at the date of consent Registered on the kidney transplant list Recipient of a deceased donor kidney only Able and willing to provide written informed consent Able to comply with study requirements On dialysis at the date of consent Donor age >10 years
Exclusion criteria	1. Evidence of HIV, HCV or current HBV infection at screening^11^The last available result from the referring unit will be acceptable2. Multiorgan transplant or a previous non-renal transplant3. Planned ABO-antibody or HLA-antibody-incompatible transplant^2^ or patients with delisted HLA specificities^2^Participants may be considered eligible and randomised into the trial before cross-matching results are known. This will allow sufficient time for randomisation to take place in cases where results of cross-matching are delayed. However, the IMP should not be removed from the freezer until it is known that the transplant is going to take place.4. Recipient of a living-donor kidney5. Recipient of a DCD kidney Maastricht category 1 or 26. Paediatric-en-bloc or an adult double-renal transplant7. Kidneys undergoing machine perfusion both ex vivo and normothermic regional perfusion8. Pregnant or lactating patients (females of childbearing potential (FOCBP)^3^ with a positive serum pregnancy test at screening)^3^FOCBP are females who have experienced menarche and are not surgically sterilised (eg, hysterectomy, bilateral salpingectomy or bilateral oophorectomy) or postmenopausal. Postmenopausal state is defined as no menses for 12 months without an alternative medical cause. A high follicle-stimulating hormone (FSH) level in the postmenopausal range may be used to confirm a postmenopausal state in women not using hormonal contraception or hormonal replacement therapy. However, in the absence of 12 months of amenorrhoea, confirmation with more than one FSH measurement is required.9. Female patients of childbearing potential who are not willing to use a highly effective method of contraception^4^ for at least 1-month post-transplantation to prevent pregnancy, or abstain from heterosexual activity.^4^Highly effective methods of contraception are those with a failure rate of <1% per year when employed consistently and correctly, for example, Combined (oestrogen and progestogen containing) hormonal contraception associated with inhibition of ovulation—oral, intravaginal, transdermal Transdermal progestogen-only hormonal contraception associated with inhibition of ovulation—oral, injectable, implantable Intrauterine device Intrauterine hormone-releasing system Bilateral tubal occlusion Vasectomised partner, provided that partner is the sole sexual partner of the FOCBP trial participant and that the vasectomised partner has received medical assessment of the surgical success 10. Male patients who are not willing to use an effective method of contraception (condoms), at least 1-month post-transplantation, when engaging in sexual activity with an FOCBP11. Involvement in other interventional trials from the date of consent until completion of visit week 5212. Known hypersensitivity to Mirococept and/or its excipients^55^Key excipients: L-arginine and D-mannitol13. Receiving treatment with a systemically administered complement inhibitor^66^eg, intravenous eculizumab for atypical haemolytic uraemic syndrome

DCDdonation after circulatory deathHBVhepatitis B virusHCVhepatitis C virusHLAhuman leucocyte antigenIMPinvestigational medicinal product

The length of time on the waiting list is variable. This could range from weeks to months during the planned 1-year and 4-month recruitment period. Therefore, following the offer of a donor kidney and on admission to hospital for transplantation, potential participants will be offered the PIS again by a member of the direct care team during the surgical work-up period serving as a reminder of the trial and invitation should the patient not have received the PIS and PIL by post. The participant’s medical history and results will be reviewed by the clinical care team (or research team if permission has been sought from the patient via the clinical care team) to confirm eligibility for participation in the study.

Potential participants will be able to discuss the trial with the recruiting site physician, have an opportunity to ask any questions and be given time to decide during the surgical work-up period whether they want to participate. The surgical work-up period can range from a few hours to a full day. If the patient is willing to participate and has had sufficient time to decide, written informed consent will be taken prior to entering theatre.

A pre-screening log will be used to record the number of participants potentially eligible but not entered into the trial in order to fulfil Consolidated Standards of Reporting Trials reporting guidelines. A consent and enrolment log will be maintained by the site. Potentially eligible participants who decline to take part will be asked if they are willing to provide a reason, but the patient does not have to provide a reason if they do not wish to. This will be captured anonymously on the pre-screening log.

### Consent

It is the responsibility of the principal investigator (PI) or delegate at physician level at each site to obtain written informed consent for each participant prior to performing any trial-related procedures. All trial investigators seeking consent must be up-to-date with their GCP training and delegated for the task on the delegation log.

The PIS is provided to facilitate the informed consent process. Two different versions of the PIS will be used (online supplemental material)—one for potential participants of the safety run and the other for potential participants of the RCT part of the trial. This is to make clear to potential participants what their participation will involve and how their results will be used, as this differs between the safety run and RCT. The safety run PIS will only be used during the recruitment period for the safety run. Once recruitment to the safety run is complete and RCT recruitment is initiated, the RCT PIS will be the only version used. Investigators must ensure that they adequately explain the aim, trial treatment, potential risks and benefits of taking part in the trial. The patient should be given sufficient time to read the PIS and to discuss their participation with others outside of the clinical research team. The patient must be given the opportunity to ask questions which should be answered to their satisfaction. The right of the patient to refuse to participate without giving a reason must be respected.

If available, the research team will access Trust interpretation services for an interpreter to translate verbal explanations and/or written patient information. The PIS can be printed in a larger font, and/or read out loud for people who may not be able to adequately understand the written information.

If the patient decides to participate in the trial, they will be asked to initial each consent clause and sign and date the latest ethically approved version of the ICF (online supplemental material). The form must also be signed and dated by the PI or delegate. Details of the informed consent discussions should be recorded in the participant’s medical notes. A copy of the signed ICF and PIS should be provided to the participant, and a copy will be kept in their medical records. The original signed consent forms will be kept in the Investigator Site File. Written informed consent will be obtained prior to conducting any study-related procedures. With the participant’s consent, their GP (general practitioner) will be informed of their enrolment onto the trial.

The routine assessments carried out before transplantation for surgical work-up and the additional research samples being collected at Guy’s Hospital for the trial will form the screening and baseline assessments for the consented participants on the trial. The PI or their delegate will confirm the participant’s eligibility for the trial prior to enrolment/randomisation.

## Randomisation procedure/code break

### Randomisation

Recruiting site personnel will be responsible for randomisation of individual participants in the multi-arm randomised stage.

If safety is met, the remaining participants will be randomised to all doses meeting the safety criteria or placebo on a 1:1:1:1 basis, stratified by centre and donor type (there are two main types of deceased donation—DBD and DCD).

The randomisation service will be provided by King’s College London (KCL) Clinical Trials Unit (CTU).

Once ensuring all inclusion and exclusion criteria (with the exception in some cases of crossmatch results) are met and written informed consent is obtained, the participant must be registered on the MACRO trial database. Following registration of the participant on MACRO, a participant ID will be generated. The participant can then be randomised, using their participant ID, via KCL CTU’s randomisation service made accessible to all the centres via the internet (https://cturandomisation.iop.kcl.ac.uk/index).

A step-by-step guide to registering and randomising a participant will be provided to centres in their site files.

In the event of a drug kit number being assigned, and the transplant not going ahead, the pack must be quarantined in the area pre-designated for quarantined kits, until it can be reassigned for use. The coordinating centre should be informed as a matter of urgency if this situation arises so the unused drug kit can be reassigned for future use.

### Emergency code break

While the safety of participants in the trial must always take priority, maintenance of blinding is crucial to the integrity of the trial. Investigators should only break the blind when information about the participant’s trial treatment is clearly necessary for the appropriate medical management of the participant.

Should an alternative to unblinding not be identified, and if unblinding is required to optimise medical management of the participant, investigators should follow the emergency unblinding process below.

24 hours Emergency Code Break and Medical Information will be provided by the Emergency Scientific Medical Service. The investigator and treating physician will have the primary right to break the blind at any moment in case of emergency, and they will be able to unblind immediately and without delay. Each randomised participant will be provided with a card detailing code break telephone numbers and emergency contact details. Participants will be requested to carry this card with them at all times while participating in the trial.

## Withdrawal of participants

Participants have the right to withdraw from the study at any time for any reason. The investigator also has the right to withdraw participants from the study in the event of intercurrent illness, AEs, serious adverse events (SAEs), protocol violations, administrative or other reasons. It is understood by all concerned that an excessive rate of withdrawals can render the study un-interpretable; therefore, unnecessary withdrawal of participants should be avoided. Should a participant decide to withdraw from the study, all efforts will be made to report the reason for withdrawal as thoroughly as possible. A withdrawal visit should be arranged if agreed with the participant.

Withdrawn participants will be replaced with provision for a 10% drop-out rate.

In the event that the participant withdraws from the trial, the appropriate withdrawal page in the eCRF should be completed. On the withdrawal page the Investigator should record the date of the withdrawal and the reason for withdrawal. Data already collected prior to the withdrawal will be retained and this will be made clear in the PIS and ICF.

Death of a participant during the trial will be assessed by the independent DMC as soon as possible to determine whether the trial should be halted. If the cause of death of a participant is deemed unrelated to the trial, they may be replaced in the analysis by another eligible patient.

## Expected duration of trial

The end of the trial will be defined as the database being locked following all participants having made their final follow-up visit, the data entered into the database and all queries resolved.

Participant recruitment is expected to take approximately 16 months (4 months safety run and 12 months RCT stage). Follow-up will continue until the last recruited participant completes their 12-month follow-up visit.

## Trial assessments

The assessments in online supplemental table 1 all form part of the routine management of renal transplant patients (desirable assessments only required if being collected as part of routine clinical care) with the exception of the research samples being collected at Guy’s Hospital only.

### Assessment of safety

Specification, timing and recording of safety parameters

The following safety parameters will be collected at every visit.

- AEs and concomitant treatment profile.
- Vital signs to include:
  - Blood pressure, heart rate, respiratory rate, temperature, oxygen saturation, weight (required at day 0, desirable for subsequent visits) and height (only required at day 0).
- Haematology to include:
  - Haemoglobin, platelets, haematocrit, red blood cells, mean cell volume, white blood cell, lymphocyte, basophil, monocyte, neutrophil, eosinophils.
- Serum biochemistry to include:
  - Glucose, gamma-glutamyl transferase, potassium, sodium, creatinine, eGFR, total bilirubin, bicarbonate, alkaline phosphatase, phosphorus, total protein, magnesium, AST (aspartate aminotransferase)/ALT (alanine aminotransferase), albumin, calcium
- Urinalysis to include:
  - Protein, specific gravity, glucose, pH, ketones, leucocytes, nitrites, urine sediment.
- Urine microscopy and culture.
- Tacrolimus blood levels.

The Investigator will review all laboratory reports and will sign and date them on the day of review. If there are any findings outside the normal range, the investigator will confirm whether the result is clinically significant or not. Any laboratory report indicated as being clinically significant will be recorded on the AE section of the eCRF.

AEs will be recorded from consent up until the end of a participant’s 12-month follow-up period. All SAEs will be followed-up for a further 30 days after the last participant’s final 1-year follow-up visit date or until resolution.

All SAEs, serious adverse reactions and suspected unexpected serious adverse reactions (SUSARs) will be reported immediately (and certainly no later than 24 hours) by the Investigator to the King’s Health Partners-Clinical Trials Office (KHP-CTO) and chief investigator (CI) for review in accordance with the current pharmacovigilance policy. The KHP-CTO will report SUSARs to the relevant ethics committee and to the Medicines and Healthcare products Regulatory Agency (MHRA).

Reporting timelines are as follows:

- SUSARs which are fatal or life-threatening must be reported not later than 7 days after the sponsor is first aware of the reaction. Any additional relevant information must be reported within a further 8 days.
- SUSARs that are not fatal or life-threatening must be reported within 15 days of the sponsor first becoming aware of the reaction.

The CI and KHP-CTO (on behalf of the co-sponsors) will submit a Development Safety Update Report relating to this trial IMP to the MHRA and Research Ethics Committee (REC) annually.

All serious and non-serious AEs will be recorded in the eCRF from the time of consent including those listed above not being reported to the Sponsor.

Mirococept is administered to the kidney ex vivo and is cytotopic, very little is expected to enter the systemic circulation. In the Phase I study, doses up to 100 mg given systemically were tolerated well. The conclusions of that Phase I study (Ref: ME0579 (APT070/1)) have been summarised in the Background and are described in more detail in both the IB and Investigational Medicinal Product Dossier.

In the present protocol, we do not expect more than about 20% of the maximum dose administered to the graft to reach the systemic circulation. At the doses proposed, this estimated exposure lies in the range of 12–36 mg of drug. Therefore, we do not consider drug-related AEs to be likely.

#### Premature termination of the trial

The trial may be prematurely discontinued by the Sponsor, CI or Regulatory Authority on the basis of new safety information or for other reasons given by the Data Monitoring and Ethics Committee/TSC regulatory authority or ethics committee concerned.

If the trial is prematurely discontinued, active participants will be informed and no further participant data will be collected. The Competent Authority (MHRA) and Research Ethics Committee will be informed within 15 days of the early termination of the trial.

## Statistics

EMPIRIKAL-2 is an operationally seamless adaptive design to evaluate safety and superiority of Mirococept in reducing DGF in deceased donor kidney transplantation, as compared with placebo. The trial is randomised, double blind and placebo controlled to minimise bias.

### Sample size

Nine patients will be needed for the safety run. Allowing for a 10% drop-out rate, the target sample size will be 10 patients for this stage.

For the main multi-arm randomised placebo-controlled stage, the required sample size was calculated using a selection theory approach as proposed by Simon *et al*.^26^ Software from the Centre for Clinical Research and Biostatistics, Hong Kong was used: (https://www2.ccrb.cuhk.edu.hk/stat/phase2/Randomized.htm).

The parameters for this calculation are p=0.30, D=0.20 and k=4, where p is the estimated percentage of the lowest response rate among all treatments, D is the difference in response rate between the best dose of Mirococept and all the other treatment groups including placebo, and k is the number of treatment arms.

A sample size of 144 (36 in each arm) will be required to correctly identify the best dose of Mirococept with 90% probability. 30 patients per arm will be required if two of the proposed doses are well-tolerated and 20 patients per arm if only one dose is well-tolerated in the safety run. Allowing for a 10% drop-out rate, the target sample size for a 4-arm study will be 160 participants (40 in each arm).

Overall, up to 170 participants are needed for the safety component and main randomised study.

A sample size of 40 per arm will produce a two-sided 95% CI for the difference in population proportions with a precision that is equal to ±21.1% when the estimated DGF rate in the treatment arm is 30% (vs 50% in placebo).

### Randomisation

If safety is met, the remaining participants will be randomised to all doses or placebo on a 1:1:1:1 basis, stratified by centre and donor type. Participants observed in the safety run will not be included in the analysis.

### Analysis

Analysis will occur at the end of the trial comparing differences in DGF proportions between each dose and the control along with CIs. Safety assessments during the randomisation stage will include routine renal function and urine tests, with further investigation (eg, renal ultrasound and Doppler) as appropriate and complement activity and Mirococept levels as per the current protocol.

In the event that DGF rate and safety outcomes are similar in more than one dosing group, the final decision on which dose to take forward to the pivotal Phase III clinical trial will include the secondary outcome measures. Of these, the most important are: functional DGF rate (where the fall in creatinine is less than 10% per day for three consecutive days); and AUC based on the drop in serum creatinine level in the first 2 weeks after transplantation where the patient does not require postoperative dialysis. If the combined primary and secondary endpoints of efficacy and the safety outcomes do not discriminate between two doses, then the lower of the two doses would be taken forward to the next trial.

This would be a confirmatory Phase III trial (to be separately funded) with two arms—the best-selected dose from the current trial vs the placebo control—each of which requiring 124 patients. Differences in DGF rates would be compared between the treatment and control arms using logistic regression adjusting for stratification factors.

### Effect size

Reduction in DGF from 50% to 30% (ie, a 20-point reduction; 40% relative reduction), control versus treatment, is predicted. The base rate of 50% is modelled on Guy’s Hospital audit data over the past 3 years and consistent with DGF rates of 60% and 40% reported for DCD and DBD, respectively, in a Cambridge study of 525 consecutive kidney transplants.^27^ The estimated 20%-point reduction is justified by a life-sustaining rat isograft model in which the frequency of non-functioning grafts was reduced from 73.3% in the control arm to 36.4% in the treatment arm.^20^ Rat donor kidneys with IGF had more rapid recovery than controls, with a relative reduction in AUC of creatinine during week 1,^20^ justifying use of this measure as a secondary endpoint in the trial. A clinical trial of ex vivo normothermic perfusion in DCD kidneys was powered for a 15%-point reduction in DGF (from 50% to 35%; a 30% relative change) with power of 80% and statistical significance of α=0.05, that is, within range of the predicted effect size.^28^

## Trial Management Group

The Trial Management Group (TMG) will include the CI, statisticians, clinical trial manager, chief scientific investigator, Senior Post Doctoral Research Associate, clinical research associate and trial staff. The TMG will be responsible for overseeing the trial and will be responsible for the day-to-day management of trial activities.

The TMG will meet regularly to discuss any trial-related activities or issues and will send updates to PIs and their teams. The TMG will review recruitment figures, SAEs and substantial amendments to the protocol prior to submission to the REC and/or MHRA. All PIs and sites will be kept informed of amendments or any trial-related developments.

## Trial Steering Committee

The TSC will provide advice for the conduct of the trial. It will comprise an independent chair and other members—both independent and non-independent. Details of the TSC, frequency of meetings, its members and terms of reference will be described in the TSC charter. The TSC provides overall supervision of the trial and ensures that it is being conducted in accordance with the principles of GCP and the relevant regulations.

The TSC members will meet to discuss trial status, recruitment progress and any other relevant issues and provide recommendations to the TMG/Sponsor.

The TSC will comprise both independent and non-independent members. The TSC Chair will be independent.

## Data Monitoring Committee

A DMC will be constituted prior to the study opening. The role of the DMC is to provide independent advice on data and safety aspects of the trial. The DMC charter will detail membership and terms of reference. The DMC members will be independent and supported by the trial statistician. In order to ensure patient safety throughout the conduct of the trial, the DMC will review and evaluate accumulated safety data, study conduct and progress. The DMC will make the decisions about the continuation, modification or termination of the study.

The DMC members will convene at defined time points stated in the DMC charter.

In addition, during the safety run, a DMC meeting will be convened after each dosing cohort to review safety data and before dose escalation to the next dose is permitted.

A DMC meeting would also be triggered if any of the safety events below occur:

- More than one of three occurrences of non-perfusion (day 0/day 1) in each dosing group, or two occurrences in a row between groups, during the safety run, in the absence of technical failure or hyperacute rejection.
- Two or more patients per dosing group with DGF lasting more than 2 weeks during the safety run.
- Death of a participant.
- Or at any other time deemed necessary by the DMC chair.

## Direct access to source data and documents

The Investigators will permit trial-related monitoring, audits, REC review and regulatory inspections by providing the Sponsors, Regulators and REC direct access to source data and other documents (eg, participants’ source data worksheets, blood test reports, radiology reports, histology reports).

## Quality assurance

Monitoring of this trial will be to ensure compliance with GCP and scientific integrity will be managed and oversight retained by the KHP-CTO Quality Team.

Regular data cleans of the eCRF will be carried out by the CI team as per a Data Discrepancy Management Plan to identify any discrepancies not picked up by the eCRF validation system.

## Data handling

**Figure 2:**
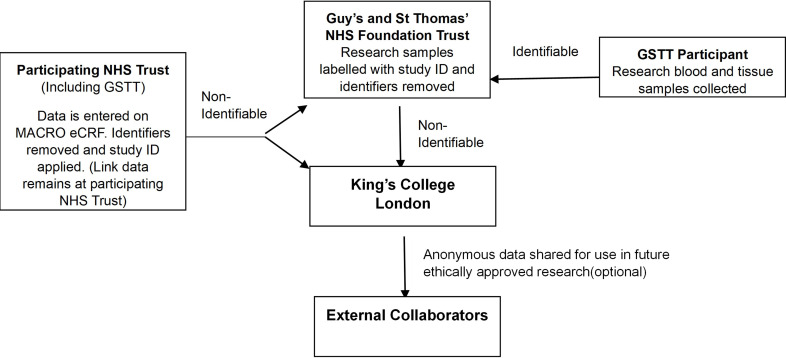
Data flow. eCRF, electronic case report form; GSTT, Guys and St Thomas’ NHS Foundation Trust.

The CI will act as custodian for the trial data (figure 2). The following guidelines will be strictly adhered to:

- Participant data will be pseudo-anonymised.
- All pseudo-anonymised data will be stored on a password-protected computer.
- All trial data will be stored in line with the Medicines for Human Use (Clinical Trials) Amended Regulations 2006 and the Data Protection Act and archived in line with the Medicines for Human Use (Clinical Trials) Amended Regulations 2006 as defined in the KHP-CTO archiving SOP.

## Data management

A study-specific data management plan has been created for this trial (online supplemental material). The data collected for this trial is a combination of routine clinical and laboratory test results commonly used in the management of kidney transplant patients and the research samples being collected for the participants at Guy’s Hospital.

### Data collection

The delegated site staff will collect data at each visit as per the schedule of events in online supplemental table 1. Source data will be collected on source data worksheets and through other source documentation. The delegated site staff responsible for data entry will be trained in the use of the eCRF system. Data entered in the eCRF system must be consistent with source data.

Sites must retain all participant records and source data for the duration of the trial for source data verification purposes during monitoring visits. All original signed informed consent forms should be filed in the Investigator Site File.

All applicable fields in an eCRF page should be completed and if data are not available, this should be clearly indicated on the form. The eCRF platform automatically creates a protected audit trail for all data entries and changes. Amendments to eCRF data will be recorded in the audit trail with a time and date stamp, along with a user-specified reason for the implemented change.

The PI may delegate data entry into the eCRF but is ultimately responsible for submitting a complete set of eCRFs for each enrolled participant.

Any supportive paper documentation (including details of any SAE) transmitted from the investigators to the Sponsor should be clearly marked with the trial name and participant trial identifier and age. Any personal information, including the name of the participant, should be removed or rendered illegible to preserve individual confidentiality.

### eCRF database

A web-based eCRF system will be designed using the MACRO system created in collaboration with the trial analyst/s and the CI team and maintained by the KCL CTU for the duration of the project. It will be hosted on a dedicated secure server within KCL.

Access to the trial eCRF platform will be password protected and electronic login credentials will be issued only to authorised individuals. It is a legal requirement that passwords to the eCRF are not shared, and that only those authorised and delegated to access the system are allowed to do so. If new staff members join the study, a personalised username and password should be requested via the Trial Manager, and a request for access to be revoked must be requested when staff members leave the project. Study site staff experiencing issues with system access or functionality should contact the CI or delegate (Trial Manager) in the first instance.

At the end of the study, once all the data reported on the database has been monitored and cleaned, the eCRF will be locked.

## Publication policy

The results of the study will be reported and disseminated at international conferences and in peer-reviewed scientific journals. Once published, a lay summary of the results will be made available to participants who request this information.

## Insurance/indemnity

This study is co-sponsored by KCL and Guys and St Thomas’ NHS Foundation Trust (GSTT). The co-sponsors will, at all times, maintain adequate insurance for the design, management and conduct of the study: (1) KCL through its own professional indemnity (Clinical Trials) and no-fault compensation policy; and (2) GSTT through NHS Resolution cover, in respect of any claims arising as a result of negligence by its employees, brought by or on behalf of a study participant.

## Discussion

DGF affects up to 50% of kidney transplants and is associated with higher rates of acute rejection, reduced graft and patient survival and prolonged hospitalisation. The incidence of DGF has significantly increased over the last two decades, probably due to the increased proportion of DCD and ECD kidneys that are being transplanted. There is an unmet need to treat DGF to improve short- and long-term graft outcomes. Local manipulation of the complement system at the site of its activation has emerged as an attractive and promising target. The lack of efficacy of previous studies with systemically administered agents might be explained by their inability to inhibit complement activation in the renal tubule. The ‘painting’ of the donor kidney before transplantation with Mirococept, a cytotopic analogue of CR1, has the potential to deliver sufficient complement inhibition with minimal systemic side effects.

Following the EMPIRIKAL trial, to our knowledge, this is the second clinical trial to implement this innovative method of ex vivo administration of a cytotopic complement inhibitor (Mirococept) in renal transplantation.

## Ethics and regulatory approvals

Ethics and Health Research Authority (HRA) approval for the study across all centres was given on 13 May 2024. The REC review was conducted by the Northeast - Newcastle and North Tyneside 2 Research Ethics Service Committee, REC reference 24/NE/0071. Clinical Trial Authorisation was issued by the MHRA on 21 May 2024. Any subsequent protocol amendments will be submitted to the REC, HRA and Regulatory Authorities for approval as appropriate.

The trial will be conducted in compliance with the principles of the Declaration of Helsinki (1996), the principles of GCP and in accordance with all applicable regulatory requirements including but not limited to the Research Governance Framework and the Medicines for Human Use (Clinical Trials) 2004, amended in 2006 and any subsequent amendments. The study is being conducted with a TMG, monitored by an independent DMC and overseen by a TSC.

Prior to site recruitment, the CI/PI or designee must receive local site approvals, for example, NHS permission in writing from the Trust Research and Development known as confirmation of capability and capacity.

The CI will submit a final report at the conclusion of the trial to the KHP-CTO (on behalf of the Sponsor) and the REC within the timelines defined in the Regulations. The KHP-CTO or delegate will upload the final report to a publicly registered database on behalf of the Sponsor.

### Trial status

The trial has been approved by the MHRA, REC and HRA. Recruitment of the first patient in the safety run was on 3 December 2024. The protocol was written in line with Standard Protocol Items: Recommendations for Interventional Trials guidelines (online supplemental material).

### Availability of data and materials

The full trial protocol is available on request to the corresponding author.

The trial team is responsible for the final trial data set. This data set and the statistical code can be made available on reasonable request after consideration by the TMG.

## supplementary material

10.1136/bmjopen-2024-097029 online supplemental file 1

10.1136/bmjopen-2024-097029 online supplemental file 2

10.1136/bmjopen-2024-097029 online supplemental file 3

10.1136/bmjopen-2024-097029 online supplemental file 4

10.1136/bmjopen-2024-097029 online supplemental file 5

10.1136/bmjopen-2024-097029 online supplemental file 6

10.1136/bmjopen-2024-097029 online supplemental file 7
