# Supplementary material for: Multicentre, multi-arm, double-blind randomised placebo-controlled dose-finding trial investigating the safety and Efficacy of MirococePt (APT070) In Reducing delayed graft function In the Kidney ALlograft (EMPIRIKAL-2): study protocol for a randomised controlled trial
Source: BMJ Open. 2025 Mar 6;15(3):e097029. doi: 10.1136/bmjopen-2024-097029 (PMC11887295; doi:10.1136/bmjopen-2024-097029)
Supplement: online supplemental file 1 [file bmjopen-15-3-s001.pdf]

DATA MANAGEMENT PLAN

A multi-centre, multi-arm, double-blind randomised placebo-controlled dose finding trial investigating the safety and Efficacy of MirococePt (APT070) In Reducing delayed graft function In the Kidney Allograft (EMPIRIKAL-2)

IRAS Number: 1008476

APPROVAL PAGE

Version number	V1.0
Date	22/10/2024
Lead Sponsor	Guy’s and St Thomas’ NHS Foundation Trust (GSTT)
Co-Sponsor	King’s College London (KCL)
Chief Investigator	Dr Theodoros Kasimatis

22-Oct-2024

Author: Lilia Dela Cruz, Clinical Data Manager

Date *(dd-mmm-yyyy)*

22-Oct-2024

This document has been reviewed by:
Alima Rahman, Clinical Trial Manager (CTM)

Date *(dd-mmm-yyyy)*

22/10/2024

This document has been approved by:
Dr Theodoros Kasimatis, Chief Investigator

Date *(dd-mmm-yyyy)*

22-Oct-2024

–
This document has been approved by:
Dr Abdel Douiri, Trial Statistician (Lead)

Date *(dd-mmm-yyyy)*

22-Oct-2024

This document has been approved by:
Mariusz Racz, Clinical Research Associate

Date *(dd-mmm-yyyy)*

CHANGE HISTORY

Version Number	Version Date	Affected Section(s)	Summary of Revisions Made:
1.0	22OCT24	N/A	Initial Release

Contents

APPROVAL PAGE 2
CHANGE HISTORY 3
GLOSSARY OF TERMS AND ABBREVIATIONS 5
1. PURPOSE 6
1.1 Data Management Plan Objectives 6
2. STUDY BACKGROUND & DESIGN 6
3. STUDY PERSONNEL 7
4. ROLES & RESPONSIBILITIES 8
4.1 RACI Chart 8
5. VERSION CONTROL 10
6. RANDOMISATION AND INTERVENTION MANAGEMENT SYSTEM 10
7. CRF DESIGN 10
7.1 eCRF Software 10
7.2. eCRF Development 11
7.3 Database Validation 11
8. DATABASE BACKUP AND RECOVERY 11
9. CHANGES TO THE PRODUCTION EDC DATABASE 12
10. DATA ENTRY 12
10.1 Source Data Workbook/Worksheets (SDW) design 13
10.2 eCRF User Access 13
9.3 eCRF Training 13
9.4 eCRF Completion Guidelines 13
11. DATA PRIVACY 14
12. PROTOCOL DEVIATION 14
13. eCRF QUERIES 14
13.1 Validation and Raising Queries 14
13.2 Query Resolution 15
14. LABORATORY TESTS 15
14.1 Translational Research samples 15
14.2 Clinical Blood Tests including Safety Run Tests 15
14.3 Laboratory Normal Ranges 16
15. DATA ANALYSIS 16
15.1 Query Generation 16
15.2 Query Resolution 16
15.3 Data Storage 16
15.4 Reports 16
16. SAE/SUSAR REPORTING 17
16.1 SAE Reconciliation 17
16.2 Data Monitoring Committee (DMC) 17
17. QUALITY ASSURANCE 17
17.1 Automated Edit Checks/ Data Validation 18
17.2 Source Data Verification 18
17.3 Manual Data Review 18
18. DATABASE LOCK 19
19. DATA EXTRACTION AND TRANSFER 20
20. DATA ARCHIVING 20
21. RELEVANT SOPs/GUIDELINES 20
APPENDIX 1: 21
EMPIRIKAL-2 Data Entry and Cleaning Flow Diagram 21
APPENDIX 2: KHP-CTO DATABASE LOCK AUTHORISATION FORM 22
APPENDIX 3: DATA EXTRACT FORM 23

GLOSSARY OF TERMS AND ABBREVIATIONS

Term	Definition
AE	Adverse Event
CI	Chief Investigator
CDM	Clinical Data Manager
CFR	Code of Federal Regulations
CRA	Clinical Research Associate
CRF	Case Report Form (paper or electronic)
CTM	Clinical Trial Manager
DGF	Delayed Graft Function
DM	Data Management
DMC	Data Monitoring Committee
DMP	Data Management Plan
DSUR	Development Safety Update Report
eCRF	electronic Case Report Form
EDC	Electronic Data Capture
EPIC	GSTT's Electronic Health Records System
FDA	Food and Drug Administration
GCP	Good Clinical Practice
GDPR	General Data Protection Regulation
GSTT	Guy's & St Thomas NHS Foundation Trust
KCH	King's College Hospital
KCL	King's College London
KCTU	King's Clinical Trials Unit
KHP	King's Health Partners
KHP-CTO	King's Health Partners Clinical Trial Office
MACRO	eCRF system
MedDRA	Medical Dictionary of Regulatory Affairs. Dictionary for coding adverse events, medical history and physical exam findings
Mirococept	Protein agent engineered with a myristoylated peptide tail
PI	Principal Investigator
QC	Quality Control
SAE	Serious Adverse Event
SAP	Statistical Analysis Plan
SDV	Source Data Verification
SOP	Standard Operating Procedure
Source Data Location List	List of location of source documents and the associated source data
SDWs	Source Data Worksheets
TMF	Trial Master File
TMG	Trial Management Group
TS	Trial Statistician
TSC	Trial Steering Committee
UAT	User Acceptability Testing

1. PURPOSE

The purpose of this document is to describe the plan of action for all Data Management (DM) activities for the **EMPIRIKAL-2** study. This plan also identifies the documents and deliverables which will be produced as part of the DM activities.

Updates to this document should be maintained throughout the life of the trial to reflect any changes in data management procedures.

1.1 Data Management Plan Objectives

1) To outline the principle procedures/processes of KCL/GSTT Data Management (DM) for this study.

2) To indicate the documentation that must be in place to ensure consistent and efficient management of all data in the study.

3) To identify and define the study personnel and roles involved with decision making, data collection and data handling.

4) To ensure DM processes are described and defined from study initiation until database lock and archiving.

5) To contain detailed working practices related to each aspect of data management that are sufficient to allow procedures to continue to be followed in the absence of the person usually performing those responsibilities.

2. STUDY BACKGROUND & DESIGN

EMPIRIKAL-2 is a multi-centre, multi-arm double-blind randomised placebo-controlled dose-finding trial with an operationally seamless adaptive design to evaluate safety and superiority of Mirococept in reducing DGF in deceased donor kidney transplantation as compared to placebo.

An initial safety run will be conducted at Guy's Hospital to assess tolerance of the study drug with each of the three proposed Mirococept doses (60,120 or 180 mg) before the randomised controlled arm of the trial begins. 9 participants will be enrolled into the safety run and followed up for 12 months. Recruitment within each dosing group will be staggered so that the interval between participants will be a minimum of 48 hours and up to two weeks. In between the dosing groups, there will be a review of safety data before dose escalation to the next level. The participants observed in the safety run will not be included in the analysis.

If safety is met, 144 participants (36 per arm) (allowing for a 10% drop out rate) will be randomised to all doses meeting the safety criteria on a 1:1:1:1 basis, stratified by centre and

donor type (There are two main types of deceased donors – Donors after Brain Death (DBD) and Donors after Cardiac Death (DCD)).

90 participants (30 per arm) will be required if 2 of the proposed doses are well-tolerated and 40 participants (20 per arm) if only one dose is well-tolerated in the safety run.

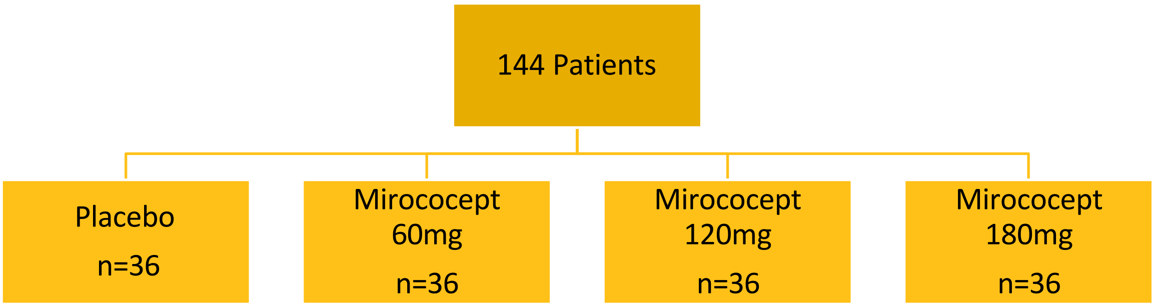

Allowing for a 10% drop out rate, the target sample size for a 4-arm study will be 160 participants (40 in each arm). Overall, up to 170 participants are needed for the safety component and main randomised study.

Please refer to the Clinical Trial Protocol for further information.

3. STUDY PERSONNEL

The key team members for this study are listed below:

Name	Title	Email
Dr Theodoros Kasimatis	Chief Investigator	theodoros.kasimatis@gstt.nhs.uk
Professor Abdel Douiri	Lead Statistician	abdel.douiri@kcl.ac.uk
Alima Rahman	Clinical Trial Manager	alima.rahman@gstt.nhs.uk
Trial Management Group (TMG)	Various roles including co-investigators and research nurse	N/A

4. ROLES & RESPONSIBILITIES

The Clinical Data Manager (CDM), with the support of the Clinical Trial Manager (CTM), is responsible for creation and maintenance of the Data Management Plan (DMP) during the course of the trial. The CDM will ensure a DMP is in place before data collection begins.

The Chief Investigator (CI) will act as custodian for the trial data. The CI has oversight of the data management processes and should, in discussion with the CTM, delegate where appropriate any ad hoc data management activities.

The CTM and CDM are responsible for the data cleaning of the study data and the Clinical Research Associate (CRA) for the monitoring of trial data.

The database will not be finalised, locked, and/or archived unless all project specific procedures have been approved by the CI. Details about all personnel involved in data management can be found in the EMPIRIKAL-2 delegation log. Specific roles and responsibilities of personnel involved in Data Management is outlined in the table below:

4.1 RACI Chart

- **Responsible:** Staff that perform the task and is responsible for recommendation, action and implementation.
- **Accountable:** Staff that are accountable for ensuring the task aligns with the study protocol and ensures the quality of the task.
- **Consulted:** Staff that provide input and/or insight prior to any actions or recommendations made.
- **Informed:** The staff that need to be informed when an action is taken or a decision is made.

Task	CDM	CTM	Database Developer	Site/ Research team	Trial Statistician	Sponsor/ CRA	CI	PI
Data Management Plan	R	C		I	C	C	A	
eCRF development and update	C	R	R	C	C	C	C/A	I
Source Data Workbook	R	A		C	I	C	A	I
Database Validation & User Acceptance Testing	R	R	R	R/C	R/C	C	A	
Data entry				R				A
Data monitoring	R	R/C		I	I	R/A	I	I
Data Discrepancy Management (remotely/centrally)	R	R/C		I	I	C	A	I
Database support		I	R/A					
Data Quality	R	R		R	C	C	A	A
Database lock	I	C/I	R		C	C	A/C	C
Data extraction	I	C	R	I	C	I	C	I
Data analysis	I	I		I	R	I	A	
Development Safety Update Report (DSUR)		C		I		A	I	I
Data reporting (Independent Data Monitoring Committee (IDMC), Final Study Report (FSR)		I			R		A	
Data Archiving	I	C		C		R	A/I	I

5. VERSION CONTROL

This document should be used as an overview for procedures undertaken within the EMPIRIKAL-2 study and should not be used in isolation.

The DMP should be managed within a version control system. A list of changes from all previous documents will be kept. The list will be cumulative and identify the changes from the preceding CRF DESIGN

The CRF design will incorporate all specifications required by the study protocol. The CI is required to approve both the Source Data Worksheets (SDWs) and electronic CRF (eCRF).

The final SDWs and eCRF will be available before the first patient is enrolled into the study.

6. RANDOMISATION AND INTERVENTION MANAGEMENT SYSTEM

A web based randomisation system using the bespoke King's Clinical Trial Unit (KCTU) randomisation system will be used. The system will be used for randomising participants and tracking study interventions. No personal details of the participant's other than initials and year of birth will be entered onto the randomization system.

Following registration of the participant on MACRO (eCRF), a participant ID will be generated. The participant can then be randomised, using their participant ID, via KCL CTU's randomisation service made accessible to all the centres via the internet (https://cturandomisation.iop.kcl.ac.uk/index)

7. CRF DESIGN

The eCRF design will be designed and produced by members of the study research team and will incorporate all specifications required by the study protocol.

The final eCRF will be available before the first patient is enrolled into the study.

Please refer to the KHP – CTO/CT/SOP8.0 Case Report Design.

7.1 eCRF Software

The software model selected for the eCRF and data entry processes for EMPIRIKAL-2 study is InferMEd MACRO 4.

InferMEd MACRO is a software solution provided by KCTU. It is an Electronic Data Capture (EDC) system, it is fully validated and is compliant with 21 CFR FDA Part 11 and Good Clinical Practice (GCP).

InferMed will be responsible for the database build system validation and hosting the data. The database has been built using MACRO™ Electronic Data Capture version 4.0. The hosting solution provides a secure, validated environment and is run in partnership with hosting company Rackspace®.

7.2. eCRF Development

The eCRF design will incorporate all specifications required by the study protocol and as indicated in the specification pack.

The CI and the Trial Statistician(TS) are required to approve the eCRF, including system edit checks and custom functions, prior to the database moving into production. Details of all approvals should be filed in the Trial Master File (TMF).

The development process for the eCRF should be done in compliance with KHP-CTO/CT/SOP8.0 Case Report Design, KHP-CTO-CT/SOP15.0 Clinical Trial Computer System Validation and KHP-CTO/CT/SOP18.0 Data Management in Clinical Trials.

7.3 Database Validation

The CI will be responsible for validation of system setup. The CI, TS, CTM, CDM and other research team members will conduct and document results of User Acceptability Testing (UAT).

Users will be given access to a test eCRF for training and UAT purposes. Each study specific implementation of the eCRF should be tested with results being documented in a test log.

Once all stages of Development and the eCRF have passed UAT, all documentation produced during this stage is signed off by the CI and TS, and the system has been validated by the KCTU, the database can be moved to production 'Go-live' status.

Any changes to the eCRF system after production must be pre-approved by the CI and Statistician before sending to KCTU.

The validation process for the eCRF should be done in compliance with guideline KHP-CTO/CT/SOP 15.0 – Clinical Trial Computer System Validation.

8. DATABASE BACKUP AND RECOVERY

The Elsevier InferMed MACRO system is hosted on the KCL server and is subject to the standard KCL central IT policies, systems and processes on data back-up and disaster recovery.

9. CHANGES TO THE PRODUCTION EDC DATABASE

The maintenance or post-production support of the eCRF should be done to comply with the objectives of KHP CTO SOP 18 Data Management in Clinical Trials.

Request for changes to the eCRF may be initiated by the CTM following a protocol change or a substantial amendment. Any other post-live database change requests will need to be discussed with the CTM and CDM. If deemed necessary the CTM will request the CI and Statistician's approval.

The CI and Statistician are to approve the request by email copying in the CTM and the CDM. The CTM will notify the Database Programmer of this request by email.

The eCRF changes to the database post-production are configured and tested following the same procedures the eCRF was configured and tested during the development environment. On completion of testing of all changes, the CTM will notify the Database Developer of the results using the appropriate form by email. When all the eCRF changes have been implemented and tested, a go-live approval page will be produced and signed off by the CI and Statistician.

The Database Developer prior to proceeding with the 'Go live' of the database, will notify the CTM by email about when the changes will 'go live'. The CTM will then notify the study team of the 'Go live' date of these changes.

10. DATA ENTRY

Data entry onto the eCRF is the responsibility of trial site staff. The delegation log outlines a list of individuals authorised for data entry and query resolution All trial site staff responsible for eCRF completion will be trained appropriately; the training will be documented.

The trial site staff will create database records for every screened subject via the EDC system.

Source documents may include but are not limited to medical records, clinic notes, appointment books, patient questionnaires, laboratory results & notes, signed and dated informed consent forms, computer printouts, patient enrolment and identification log and SDWs.

Please refer to the Source Data Location List in the Investigator Site File.

10.1 Source Data Workbook/Worksheets (SDW) design

The SDW design will incorporate all specifications required by the study protocol. SDWs will be designed by the CDM following eCRF production and upon consultation with members of the study research team. They will also be reviewed by the CTM and CRA.

SDWs will be version controlled and updated in line with the protocol changes. They will be completed for all patients as source data documents. The CI is required to approve all the SDWs.

The final SDWs will be available before the first patient is consented into the study.

10.2 eCRF User Access

Each user must be trained on the eCRF prior to being granted permission to use it. All users require an account to allow them to access the eCRF. The CTM will be notified of new user requests and these will be forwarded to KCTU for action. Each user will have different access privileges to the eCRF depending on their role.

To protect the security of data in the database, user accounts are issued only if the following documents are available:

- CV and GCP certificate of the trial staff member
- Evidence of trial-specific training (e.g. attendance at the site initiation meeting or documentation of training conducted after the site initiation meeting)
- A signed delegation log of the trial staff member who requires access.

9.3 eCRF Training

Data entry onto the eCRF will be performed by designated staff that have had eCRF training. Each user must be trained on the EDC system prior to being granted permission to work on the eCRF. A record of eCRF training will be kept in the main Training Log for those that did not attend the Site Initiation Visit (SIV)and who are delegated this responsibility.

9.4 eCRF Completion Guidelines

Online training material can be found on the KCTU website. A database user guide document, with detailed instructions of data entry will be created for this study to assist the site personnel when entering data. Site staff will be requested to enter data as soon as possible and preferably within a week from data collection.

The guidelines will be revisited during the course of the study and will be updated as required and version-controlled.

11. DATA PRIVACY

All source documents, the eCRF and data export are kept within secured locations and comply with the Data Protection Act 2018 & General Data Protection Regulation (GDPR).

All delegated users of the EDC system will be registered with an account and provided password protected access. Access to the EDC system will be granted to trained users.

All data stored on the database are pseudo-anonymised and data entry personnel will ensure patient identifiable data are not included in the text fields of the data entry screens. The participants will be identified in the study database only by initials and a participant ID number.

12. PROTOCOL DEVIATION

It is a requirement to report/document any deviations that may significantly impact on the completeness, accuracy, and/or reliability of the study data.

All deviations should be recorded on the deviation log. Further details should be given for protocol deviations that require further explanation and any Corrective and Preventative Actions. This information can be used whenwriting the Clinical Study Report.

13. eCRF QUERIES

13.1 Validation and Raising Queries

System generated queries are automated edit checks generated by the EDC system on discrepant data.

The automated edits checks required for the study are discussed at the database development meetings attended by the CI, Trial Statisticians, CTM, KCTU database developers and CDM. The study team will be responsible for providing the agreed automated edits checks to the developer's team. These checks will be defined in the database specification forms.

Automated queries/warnings will be raised immediately when any out of range values are entered and will need to be resolved by confirming the entry before it can be submitted. Once the eCRFs have been source data verified by the CRA during a monitoring visit, the CRA will raise queries if required. The CDM and/or CTM will perform discrepancy management and issue additional queries on any discrepant data or where further clarification is required in relation to automated queries. Any necessary DM queries will be issued via the eCRF. A Data

Discrepancy Management Plan (DDMP) will be devised; in discussion with CI, Trial Statistician, CTM and CRA.

The trial site staff will answer the queries raised by the CRA and CDM/CTM, and update the eCRF data when appropriate and submit responses through the eCRF.

Once the eCRF forms/screens are declared clean and frozen by the monitor, the PI must complete the signature panel associated with each relevant form.

If further queries are raised and resolved after the signature has been applied, the eCRF will require re-signing by the PI.

As part of the ongoing quality control process, the CTM and CDM will monitor the type and number of queries experienced at the site on an ongoing basis. If necessary, the CTM and CRA will help to have the queries resolved and answered.

See Appendix 1.0 for Data Entry & Cleaning flow diagram.

13.2 Query Resolution

The CTM and CDM will be responsible for closing system and data management queries, while the CRA will be responsible for closing the source data verification (SDV) queries that have been answered on the eCRF. For manually opened queries, the general rule is, whoever opened the query should also review and close the query.

14. LABORATORY TESTS

14.1 Translational Research samples

The following research samples will be collected from Guy's Hospital participants only:

- serum for antibodies to Mirococept
- serum for Mirococept levels
- serum of complement activity levels (CH50)
- renal biopsy specimens (optional)
- whole blood for biomarkers (optional)

Synnovis is the laboratory used for the analysis of CH50 samples. The biomarker samples will be analysed at the Immunomonitoring Lab and the others at the Protein Therapeutics Lab within the Svhool of Immunology and Microbial Sciences School at KCL. Please refer to the EMPIRIKAL-2 Protocol and Laboratory Manual for further information.

14.2 Clinical Blood Tests including Safety Run Tests

All required laboratory investigations that form part of the routine management of renal transplant patients will follow the site's local laboratory's sample handling SOPs.

All clinical laboratory values will be entered on the eCRF by the trial site staff.

14.3 Laboratory Normal Ranges

Synnovis and sites' local laboratory reference ranges will be used for clinical assessment which will be verified by the clinician on the source documents.

15. DATA ANALYSIS

15.1 Query Generation

On receiving an extract, the Trial Statistician will do an offline edit check to ensure that the data is clean before and during analysis, i.e. frequencies will be run on the key study variables to look for unexpected results. Any discrepancies will be documented.

15.2 Query Resolution

The Trial Statistician will send the documented discrepancies to the relevant member of the research team (which may be the CI or may include the CI). Queries will be discussed with the team and resolved accordingly.

15.3 Data Storage

The CI and the Trial Statistician are responsible for requesting data extracts for this study.

All data will be stored in named and dated network files and is accessible only to the Trial Statistician.

15.4 Reports

Reports will be run at regular intervals to identify any missing forms and these will be followed up and tracked by the CTM.

Status reports will be monitored by the CTM throughout duration of the study.

The following status reports will be used during the course of the study:

- DMC reports
- DSUR reports

For each DMC meeting, the CTM and the Trial Statistician will agree the date on which a data extraction is required. This will be at least 4 weeks prior to the meeting. The Trial Statistician will submit to the CTM the data extract form with details (please see appendix 22.4). The CTM/CDM will confirm data has been exported to the Trial Statistician. The DMC Data Report prepared by the statistician will be sent to all the DMC members at least 1 week prior to a scheduled meeting by the CTM.

16. SAE/SUSAR REPORTING

In the event of a Serious Adverse Event (SAE) that is not listed in the protocol as exempt from reporting, or Suspected Unrexpected Serious Adverse Reaction (SUSAR) occurring whilst a participant is on the study; this will be reported directly to the King's Health Partners (KHP)-Clinical Trials Office (CTO). A KHP-CTO SAE form will need to be completed.

For further information please refer to the latest version of the KHP – CTO Pharmacovigilance and Safety reporting policy:

http://www.khpcto.co.uk/Documents/SOP/PV%20Policy%20Final%20v6%2011-11-13.pdf

16.1 SAE Reconciliation

SAE reconciliation of safety data will be undertaken by by the CRA.

The KHP-CTO will report SUSARs to the regulatory authority, MHRA. The CI team/ GSTT R&D team will report SUSARs to the relevant ethics committee.

16.2 Data Monitoring Committee (DMC)

The role of the DMC is to provide independent advice on data and safety aspects of the trial. The DMC charter will detail membership and terms of reference. The DMC members will be independent and supported by the trial statistician. In order to ensure patient safety throughout the conduct of the trial, the DMC will review and evaluate accumulated safety data, study conduct and progress. The DMC will make the decisions about the continuation, modification or termination of the study.

17. QUALITY ASSURANCE

Ongoing and final data quality checks will be done by the CDM/CTM, before the database is finalised.

Inconsistencies in the trial data will be investigated using data queries that prompt the trial centre to clarify or confirm discrepant items. The eCRF system will incorporate automated and manual query generation tools. The CDM with support from the CTM will systematically

check incoming trial data for consistency, omissions and compliance with the protocol. Monitoring of this trial aims to ensure compliance with Good Clinical Practice and maintaining scientific integrity. Monitoring will be managed and oversight retained by the KHP-CTO Quality Team.

To assure data quality of the EMPIRIKAL-2 study, the following steps will be performed:

17.1 Automated Edit Checks/ Data Validation

Automated or online edit checks provide the first quality control (QC) step, immediately after an eCRF page is saved as complete. These checks ensure the completeness, plausibility and consistency of manually-entered trial data. Programmed edit checks run online at the time of saving each screen/page. Data entered that violates a validation rule will automatically generate a data query which is instantly visible to the user on the eCRF. Queries can be answered immediately and, if necessary, the data in the eCRF can be changed by the user directly in the online query form.

The eCRF has inbuilt features to provide every variable with an audit trail, throughout the duration of the study, and store comments on changes to saved data (ICH E6 R2 4.9.3)

17.2 Source Data Verification

Source data verification (SDV) occurs after the automatic validation checks. It will be performed by the CRA who will visit the trial centre during site monitoring visits. SDV will ensure the accuracy of manually entered trial data by comparing eCRF entries against the source data available at the site. The extent and frequency of SDV is specified and defined in the study Monitoring Plan.

Throughout the study and prior to database lock, the sponsor CRA will confirm that all participant data has been entered and source data verified in accordance with the Monitoring Plan.

17.3 Manual Data Review

Manual review of the study data is the final QC step and will ensure consistency of trial data. It will be employed to check aspects of the dataset that cannot be checked viaautomatic validations (e.g. complex reconciliation of data across two or more eCRF pages) or to perform checks that require medical interpretation (Medical Review).

Manual data review will be undertaken by the CDM/CTM remotely using the Data Discrepancy Management Plan (DDMP). The CRA will undertake manual review by performing SDV during site visits . Medical review will be undertaken by the PI or medically qualified delegate..

18. DATABASE LOCK

Database lock only takes place at the final stage of the study when all eCRF records are completed, all queries resolved and PI sign-off for each participant. All user accesses are removed with no further changes to the database expected.

The Trial Statisticians, CTM, CDM, and CRA, in agreement with the CI, declare the database clean database lock is requested.

The database hard lock procedure follows the guidance set out by the KHP-CTO. **This lock only takes place after all study datadata activities are completed.**

It is only done upon successful completion of the following:

- All expected data is entered.
- All expected data has been received, processed and validated for completeness and consistency.
- All expected data is source verified by the CRA as detailed in the Monitoring Plan.
- All programmed and manual data checks have been performed and issues resolved.
- SAE reconciliation is complete and approved by the CI.
- All updates to the database have been completed.
- All discrepancies found during review have been queried, resolved, and the data corrected where necessary.
- Quality review of the data has been performed and checked by the statisticians.
- The PI must log on and sign off each patient's data to confirm it is accurate. This changes the data status and freezes the data.

Following this, the CTM will organise a meeting with the CI team, Statistician and CRA to confirm that all parties are happy to proceed with DB lock and understand what this involves. The KHP-CTO Database Lock Authorisation Form (see appendix) must also be completed and signed by the CI, CRA and Trial Statistician.

After the database hard lock has been agreed and authorised internally by the CI, CRA and Trial Statistician, the CTM will send a copy of the signed authorisation form to the database developer/ MACRO team - ctu@kcl.ac.uk and request for the database to be locked. The CI, TS, CRA and all other key staff should be copied in.

Please note, further changes shouldn't be applied to the database.It involves removal of access for all users.

The original copy of the signed KHP-CTO Database Lock Authorisation Form will be filed in the relevant section of the study TMF.

19. DATA EXTRACTION AND TRANSFER

The Trial Statisticians will provide a specification/script of the datasets required. These specifications will include datasets and variable names together (clearly labelled) with the data dictionary in a CSV, STATA or other named format.

The final data files will be sent as CSV, STATA or other named format datasets. The files will be sent via email as password protected zip files. The password will be sent in a separate encrypted email for transfer methods that require a password.

In addition, the sponsor may request ad hoc transfers of data at any stage during the study.

Transfer of cumulative data sets (including full documentation) will be prepared as required for electronic submission of data to authorities.

20. DATA ARCHIVING

All trial data will be stored in line with the Medicines for Human Use (Clinical Trials) Amended Regulations 2006, the Data Protection Act, and GDPR and archived in line with the Medicines for Human Use (Clinical Trials) Amended Regulations 2006.The trial database will be locked before the final analysis in accordance with the KHP-CTO/CT/SOP4.0 Archiving of Clinical Trial Data and Essential Documentation.

Prior to data archiving, all study close-out activities as set out by KHP-CTO on https://khpcto.co.uk/SOPs/16_siteCloseOutSOP.php must be completed.

21. RELEVANT SOPs/GUIDELINES

The following KHP-CTO SOPs and Working Guidance Documents will be followed:

Title
Clinical Trial Monitoring
Archiving of Clinical Trial Data and Essential Documentation
Case Report Design
Clinical Trial Computer System Validation
Data Management in Clinical Trials

APPENDIX 1:

EMPIRIKAL-2 Data Entry and Cleaning Flow Diagram

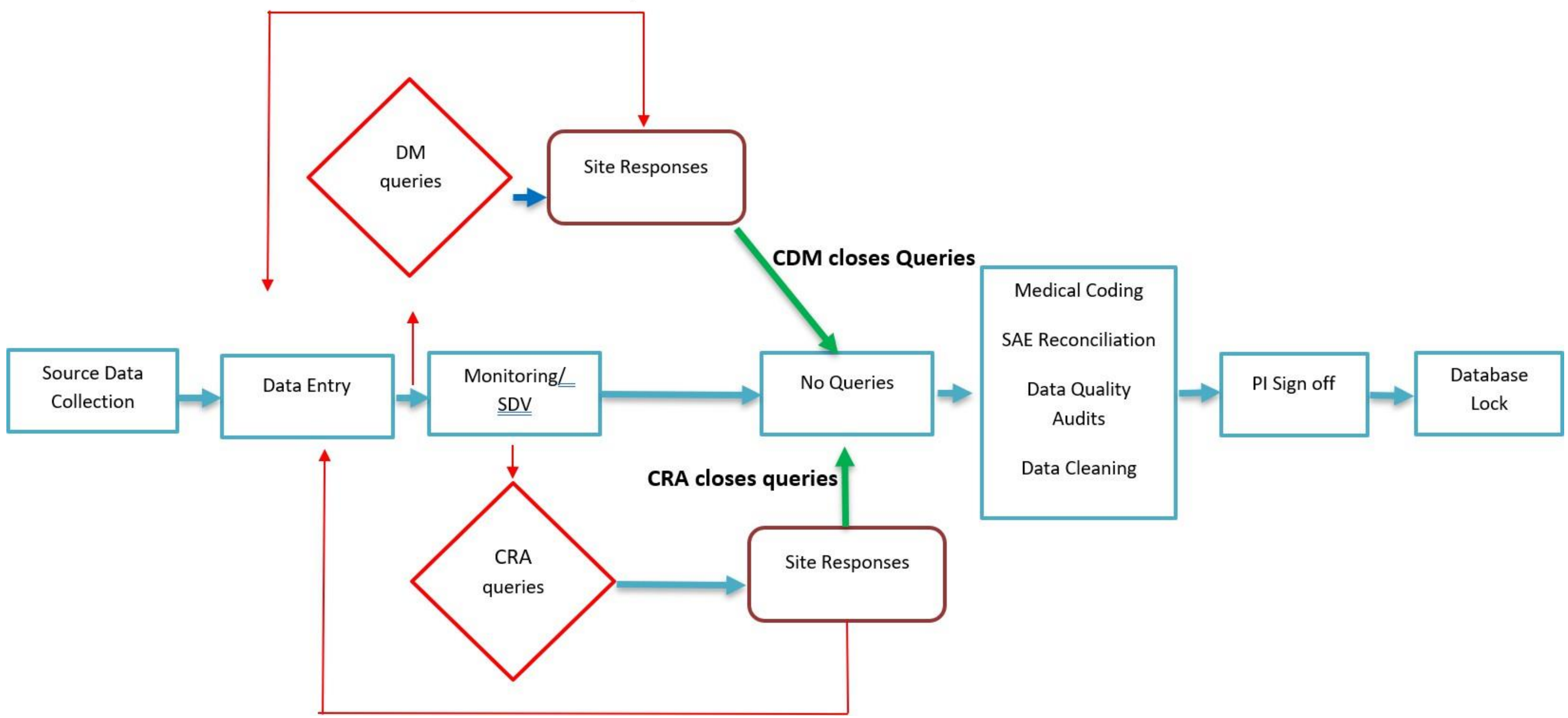

APPENDIX 2: KHP-CTO DATABASE LOCK AUTHORISATION FORM

KING'S HEALTH PARTNERS

An Academic Health Sciences Centre for London   Pioneering better health for all

King's Health Partners Clinical Trials Office
Database Lock Authorisation Form

Trial Details

**Trial Title:**	
**CI Name:**	
**(Co-)Sponsor(s):**	
**IRAS Number:**	
**MATTS Number:**	

The .... database for the .... study will be locked formally before code break, as all statistical checking has been completed.

I confirm that all data has been entered by study sites, all data queries have been raised and resolved, all discrepancies closed, IMP, SAE and emergency code break reconciliation have been completed and are reflected in the database.

I confirm that data within the database is accurate and complete, and the database is ready to be locked.

I understand that no changes to the dataset will be possible after database lock.

Date of database lock: ________________________

**Study CI Name:**		
**Signature:**		**Date:**

**Statistician Name:**		
**Signature:**		**Date:**

**Study CRA Name:**		
**Signature:**		**Date:**

APPENDIX 3: DATA EXTRACT FORM

Part A *Please ensure that if you are submitting a request you have considered any relevant blinding issues so that the blinded status of the study is not compromised. If unsure, please refer to the database codebook(s) prior to completing an extract request.* <br>*It is recommended that you have a signed off SAP in place before you make a request for any data extracts containing outcome data.*	
**Name of study:**	
**Macro version:**	☐ Macro version 4
**Individual requesting data extracts within the study team:**	☐ Chief Investigator ☐Statistician/analyst ☐ Health economist ☐ Study Trial manager ☐ KCTU Data Manager ***Please note, only these individuals can request data extracts from the CTU***
**Name of individual requesting data;**	
**Date:**	____/____/ _________

Part B – Data Extract Request Details: ***To be completed each time a data extract is required and emailed to: ctu@kcl.ac.uk for processing.***	
**What database do you require data from?**	☐ <Insert Database 1> ☐ <Insert Database 2> ☐ <Insert Database 3> ☐ <Insert Database 4>
**How would you like your data extracted?**	☐ By Visit (e.g. all data collected, exported by visit) ☐ By Form (e.g. all data collected, by each specific form across all time points)
**Specify visits/forms to be exported (as per study timeline) or tick 'all'** <br>*Please note: please check codebook and consider any blinding issues before submitting your request to us.*	☐ All ☐ List Visits (please specify which)_____________________________________ ☐ List Forms (please specify which)_____________________________________
**What format would you like your data in?** <br>*Please note: data will be sent along with a codebook which includes information about the formats and codes applied to the observations.*	☐ Comma separated variables (.csv) ☐ SAS ☐ STATA
**How would you like your data sent to you? (e.g. emailed in a zipped folder)**	☐ Emailed in a zipped folder ☐ **Other, please specify details; ____________________________________* **Please note this option should have been agreed at the contracting stage.*
**What is the purpose of the data extract?**	☐ Database development example test data extract for part 2 signoff. ☐ Baseline paper/publication ☐ DMC/DMEC/TSC Report ☐ Trial Management Reports (Ethics/Regulator/Funder/Monitoring) ☐ Data Management Report ☐ Data cleaning ☐ Central Statistical Monitoring ☐ Interim analysis ☐ Pre-analysis statistical checks ☐ End of study data extract for analysis ***(please note; before selecting this option ensure all ldata locking is complete as no further extracts will be processed beyond this point and all user access will be removed)***
Please note: if you also require data from the KCTU randomisation system please contact: ctu@kcl.ac.uk <br>***It is the responsibility of the study team to retain copies of all data exports provided throughout the life cycle of the trial***	
**Where should the data extract(s) be sent?**	
**Receiver name:**	
**Receiver email address:**	
If the data must be exported on a specific date please specify here;	____/____/ _________

Please note; All data extracts will be processed within 5 working days from the point at which the request is acknowledged by the KCTU and the exported data will be emailed back to the receiver email address listed above.
