# Supplementary material for: Multicentre, multi-arm, double-blind randomised placebo-controlled dose-finding trial investigating the safety and Efficacy of MirococePt (APT070) In Reducing delayed graft function In the Kidney ALlograft (EMPIRIKAL-2): study protocol for a randomised controlled trial
Source: BMJ Open. 2025 Mar 6;15(3):e097029. doi: 10.1136/bmjopen-2024-097029 (PMC11887295; doi:10.1136/bmjopen-2024-097029)
Supplement: online supplemental file 2 [file bmjopen-15-3-s002.pdf]

Patient Consent Form

Study Title – A multi-centre, multi-arm, double-blind randomised placebo-controlled dose finding trial investigating the safety and Efficacy of MirococePt (APT070) In Reducing delayed graft function In the Kidney ALlograft (EMPIRIKAL-2)

Centre: ______________________________ Participant ID:_______________________

Principal Investigator: _________________ IRAS ID: 1008476

Please **initial** box to indicate agreement

1. I confirm that I have received a personal copy of, and have read and understood, the _________________________Patient Information Sheet dated _____________ (Version ___) for the above study and have had the opportunity to discuss, ask questions and have had these answered satisfactorily. I have been given a copy of the Patient Information Sheet to keep.	[ ]
2. I understand that my participation in this study is voluntary and that I am free to withdraw at any time, without giving any reason, without my medical care or legal rights being affected.	[ ]
3. I give permission for the research team to access my medical records for the purposes of this research study.	[ ]
4. I understand that sections of any of my medical notes and data collected during the study may be looked at by individuals from the Sponsor (Guy's and St Thomas' NHS Foundation Trust and Kings College London), from regulatory authorities or from the NHS Trust, where it is relevant to my taking part in this research. I give permission for these individuals to have access to my records.	[ ]
5. I agree to my General Practitioner being informed of my participation in the study.	[ ]

6. I agree to my anonymous data being used for future ethically approved studies.	YES [ ]<br>NO [ ]
7. I understand that the information held and maintained by my local NHS Trust may be used to help contact me or provide information about my health status.	[ ]
8. I would like to receive a summarised version of the study results following the end of the study.	YES [ ]<br>NO [ ]
9. I agree to take part in the above study.	[ ]

10. I understand that new research blood samples will be collected from me as part of this research.	[ ]
11. (optional) I agree to the collection, processing, storage and analysis of **an additional blood sample** for use in future ethically-approved research studies.	YES [ ]<br>NO [ ]
12. (optional) I agree to the collection of transplant kidney biopsies before surgery and during surgery followed by processing, storage and analysis of these to further understand the impact of the study drug on the kidney.	YES [ ]<br>NO [ ]
13. (optional) I agree to my anonymous samples being used in ethically approved research in the future.	YES [ ]<br>NO [ ]

______________________ ________________ _________________

Name of Patient Date Signature

______________________ ________________ _________________

Name of Investigator Date Signature

When completed: 1 for patient; 1 for medical notes; 1 (**original**) to be kept in investigator Site File.
