# Supplementary material for: Multicentre, multi-arm, double-blind randomised placebo-controlled dose-finding trial investigating the safety and Efficacy of MirococePt (APT070) In Reducing delayed graft function In the Kidney ALlograft (EMPIRIKAL-2): study protocol for a randomised controlled trial
Source: BMJ Open. 2025 Mar 6;15(3):e097029. doi: 10.1136/bmjopen-2024-097029 (PMC11887295; doi:10.1136/bmjopen-2024-097029)
Supplement: online supplemental file 4 [file bmjopen-15-3-s004.pdf]

A multi-centre, multi-arm, double-blind randomised placebo-controlled dose finding trial investigating the safety and Efficacy of MirococePt (APT070) In Reducing delayed graft function In the Kidney ALlograft (EMPIRIKAL-2)

EMPIRIKAL-2

Patient Information Sheet

Principal Investigator: [Insert PI name]
[Insert PI Job Title]

We invite you to take part in a research study

- We would like to invite you to take part in a clinical trial (also called a clinical study).
- Before you decide whether you would like to take part in the trial, it's important for you to understand why the research is being done and what it would involve for you.
- Please take some time to read the information carefully, and discuss with your family, friends and doctor, if you would like to.
- Ask us, if anything is not clear, or you would like some more information.

Thank you for taking the time to consider taking part in the EMPIRIKAL-2 Study.

How to contact us:

Local research team contact/s:
Email:
Tel:

Key Information:

- You may have received some information about taking part in the first safety stage of the study testing the study drug, Mirococept. This information is about taking part in the next stage of the trial.
- Taking part is completely up to you and you can stop taking part at any time, without giving a reason. If you do not wish to take part, this will not affect the care you receive from your doctors or other health care professionals.
- We are testing a drug called Mirococept which will be given once only to the donor kidney outside of the body before transplantation.
- We are inviting patients who are waiting to receive a kidney transplant to take part in the trial.
- We would like to test the safety of Mirococept and how well it can help reduce damage to the kidney associated with transplantation.
- Mirococept is designed to treat the donor kidney. You will receive a kidney that has been treated with Mirococept before transplantation.
- Your care will continue as normal.
- We would like to collect some research blood samples, optional research blood samples for future research, and optional kidney biopsies with your permission.
- All other tests and procedures will be as per routine care.
- You will not be asked to attend any visits that are above the standard care you will normally receive.
- The potential risks and benefits are included in the information sheet.

Contents

1. Why is this study needed?

2. What is the drug being tested?

3. What type of trial is it and what are the aims?

4. How will the study work?

5. Why have I been asked to take part?

6. Do I have to take part?

7. What would taking part involve?

8. Will I be asked to provide any samples?

9. Will my samples be used in future research?

10. What are the possible benefits of taking part?

11. What are the possible disadvantages and risks of taking part?

12. Information on the Use of Data

MORE INFORMATION ABOUT TAKING PART

1. Why is this study needed?

Before a kidney can be transplanted into a patient it has to be removed from the donor and transported to the hospital of the patient receiving the kidney. During this time, the kidney is stored in a cold storage solution to reduce the damage caused by a lack of blood supply. When the kidney is transplanted into the patient and blood supply is reintroduced, the immune system reacts which can cause damage to the kidney tissue.

The study is looking to see whether a drug called Mirococept can help reduce the amount of damage that can happen when a kidney is transplanted.

2. What is the drug being tested?

The drug being tested is called Mirococept. This is a new class of drug that can stick to the donor kidney and prevent inflammation. Part of the drug blocks a protein in your body called “complement”. This “complement” protein can damage a transplant kidney when it does not have a blood supply. Mirococept has been specially designed so that it is given to the kidney and not directly to you. The protein used to build the new drug is based on a natural protective human protein that has been reproduced and modified outside the body. It works by blocking tissue damage caused by the uncontrolled activation of part of the immune system. Mirococept restores a natural control system that is lost during the transplant process.

Mirococept is given to the donor kidney after the kidney has been removed from the donor but before it is transplanted into the patient receiving the organ. Mirococept has a tail-like structure that helps to attach to the cells of the kidney. After treatment of the donor kidney, Mirococept remains in the donor organ. With this approach we aim to treat the organ rather than the patient, thereby reducing any potential side effects.

3. What type of trial is it and what are the aims?

This study is an early phase trial. It means the study drug described above is primarily being tested for safety purposes. Mirococept has been used before in 28 healthy volunteers and in 52 patients undergoing kidney transplantation. The study aims to find out whether the drug is safe and potentially effective in preventing damage to the

kidney. This will be measured by the number of patients that will need dialysis after the transplant. We are looking to explore what dose of Mirococept is most effective at doing this for further testing in a larger trial.

4. How will the study work?

We first invited nine patients to take part to assess if Mirococept at the doses being tested for this trial were safe to give to people receiving kidney transplants. Three different dose levels were tested. This stage is now complete. The data collected was reviewed by a panel of experts. They decided the study was safe to continue and the drug could be tested in the second stage of the trial.

Now we are ready to begin testing the three different doses of Mirococept in a larger group of participants for this second stage. We will compare these doses of study drug with the standard substance or placebo. The placebo substance contains no active ingredients. It ensures that the treatment is tested fairly. The group you are allocated to will be decided by chance.

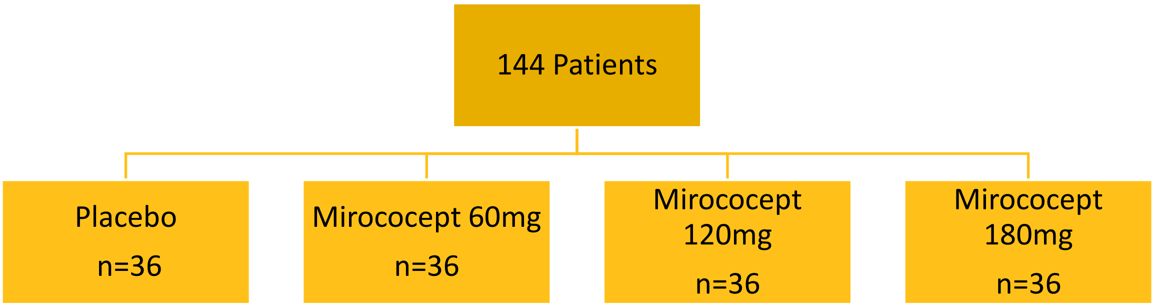

We will invite 144 patients to take part. Three quarters of the group for this second stage will receive a donor kidney that has been treated with 1 of 3 doses of Mirococept being assessed in this trial, whilst one quarter will not. The group that do not get a kidney treated with Mirococept will receive the placebo.

You are being invited to take part in the second stage of the study and be one of the 144 patients to receive Mirococept at one of three dose levels or to receive placebo.

5. Why have I been asked to take part?

You have been invited to take part in this study because you are registered on the kidney transplant waiting list and are currently waiting to receive a donor kidney. Your kidney team have assessed you to be suitable to potentially take part in this study.

6. Do I have to take part?

No, it is up to you to decide whether or not to take part. If you do decide to take part you will be given this information sheet to keep and be asked to sign a consent form. If you decide to take part you are still free to withdraw at any time and without giving a reason. Deciding not to take part or withdrawing from the study will not affect the healthcare that you receive, or your legal rights.

7. What would taking part involve?

On the day you are admitted for your transplant, we will ask you to sign a consent form to confirm that you're happy to take part in the study.

Participants will be placed into one of four groups. Three of these groups will receive a donor kidney that has already been treated with the study medication. The dose of the medication given to the kidney will either be 60mg, 120mg or 180mg, depending on which group you are in. One of the groups will receive a donor kidney that has been treated with the standard substance or placebo. There is an equal chance of being in any of the four groups. A computer will assign you to a group at random, a bit like rolling a dice. You will not know which group you have been assigned to, and neither will your doctors and nurses. However, the doctors can find out if they need to. This type of study is called a randomised, double-blind trial and it ensures that the treatment is tested fairly.

All other assessments except those listed in the table below will be in line with standard of care for all patients receiving a kidney transplant. This will include 14 visits over the course of one year once you are no longer an inpatient. You will be asked to provide research blood samples, optional blood samples for future research use and optional biopsy samples for research analysis at the times listed in the table below. We

are collecting additional research blood samples to measure the amount of the study drug found in your blood and check the effect of Mirocecept on your immune system. We are collecting optional biopsy samples to check the presence of the study drug in the donor kidney and its effect on local immune system activation.

**Sample Type**	Day 0 – pre-transplant	Day 0 – 1hr post-transplant	Day 0 – 4hr post-transplant	Day 1	Day 2	Day 4	Day 7	Week 2	Week 4	Week 10
Research blood samples	X	X	X	X	X	X		X	X	X
Future research blood sample (optional)				X		X	X		X	
Donor kidney biopsy samples (optional)	X	X								

Where possible, the blood samples will be collected alongside routine clinical blood samples to minimise discomfort. More details about these samples can be found in the next section.

We will need to inform your GP to let them know that you are taking part in the trial.

8. Will I be asked to provide any samples?

Routine blood tests will be performed as per your local hospital practice at each of your visits to the hospital.

You will also be asked for some additional blood for research purposes. This will be approximately 80ml (6 tablespoons) of additional blood whilst you are an inpatient at the times described above. These samples will be labelled by your local hospital staff using your initials and a code that can only be linked to you by the local research team at your hospital. These samples taken for research will be sent to King's College London (KCL) and Guy's and St Thomas' Hospital Laboratories (Synnovis). Your samples will not have any personal identifiable information attached to them when they are sent to KCL.

In addition to blood samples, you will be asked for kidney biopsies for research purposes. This will be optional and you can still take part in the trial if you decide you don't want kidney biopsies collected for the research. A small sample (1-2cm) of the donor kidney will be taken before treatment with Mirocecept and a second sample after the implantation of the kidney during surgery using a biopsy needle. The biopsy samples will be sent to KCL for analysis. Your samples will not have any personal identifiable information attached to them when they are sent to KCL. Your samples will either be destroyed at the end of the study via the routine process for disposal of biological samples or, may be used in future research studies with your consent.

9. Will my samples be used in future research?

We will ask for your consent for these samples to be used in future research. These samples could include the processing, storage and analysis of genetic material found in blood cells The consent for storage of samples for future research is optional and will not affect your participation in the study in any way.

These samples will be stored at KCL. None of your personal data will be shared in the event of samples being used for future research.

10. What are the possible benefits of taking part?

It is possible that Mirococept will help the recovery of your kidney function following your kidney transplant. However, we cannot say this for certain until we have completed this and future studies. You may not directly benefit from taking part in this study, but the information gained from your participation may help to improve the treatment of patients with your condition in the future.

11. What are the possible disadvantages and risks of taking part?

Risks associated with procedures

There may be bruising and discomfort at the site of the blood tests, as with any blood test. However, and where possible, the blood taken for research purposes will be collected at the same time as your routine blood tests to minimise discomfort.

There is a small risk of bleeding after a biopsy which is mostly mild and self-limiting. These biopsies will be collected before and during surgery, therefore no additional invasive procedure will be performed to collect the biopsies. They will be collected in the theatre where any potential bleeding can be quickly secured by the surgeon with a stich. The kidney biopsies are optional samples.

Risks associated with the treatment

There are unlikely to be any side effects from the drug that you will experience from taking part in this study. This is because the drug is given to the donor kidney, and very little of the drug should enter your own bloodstream. We know this from previous tests of the drug in a small number of participants. We have not experienced any such side effects in the small numbers of patients who have been treated so far, whether the drug has been given to the donor kidney directly or, infused into the bloodstream of trial participants.

However, you should be aware that any new medication can cause unexpected and serious side effects, even if these are rare. This includes potential increased risk of infection and kidney cell injury.

Harm to the unborn child

For Women:

There is no evidence that Mirococept could damage an unborn child, but this has not been tested. To be included in the study you will therefore need to have a negative pregnancy test on admission into hospital for the transplant. We do know that some of the routine transplant drugs can damage an unborn child and it is therefore common practice to require a negative pregnancy test at the time of transplantation.

Although there is no evidence that Mirococept will cause any foetal abnormalities, we require that contraception is used for at least a month following the transplant.

For Men:

There is no evidence that Mirococept could damage sperm and consequently the foetus. However, we require that contraception is used for at least a month following the transplant.

12. Information on the Use of Data

12.1 How will we use information about you?

We will need to use information from you and your medical records for this research project.

This information will include your initials, name, date of birth, NHS and hospital numbers and contact details. People will use this information to do the research or to check your records to make sure that the research is being done properly.
People who do not need to know who you are will not be able to see your name or contact details. Your data will have a code number instead. .

We will keep all information about you safe and secure.

Once we have finished the study, we will keep some of the data so we can check the results. We will write our reports in a way that no one can work out that you took part in the study.

12.2 What are your choices about how your information is used?

- You can stop being part of the study at any time, without giving a reason, but we will keep information about you that we already have.
- If you choose to stop taking part in the study, we would like to continue collecting information about your health from your hospital. If you do not want this to happen, tell us and we will stop.
- We need to manage your records in specific ways for the research to be reliable. This means that we won't be able to let you see or change the data we hold about you.
- If you agree to take part in this study, you will have the option to take part in future research using your data saved from this study.

12.3 Where can you find out more about how your information is used?

You can find out more about how we use your information:

- at www.hra.nhs.uk/information-about-patients/
- at www.guysandstthomas.nhs.uk/research/patients/use-of-data.aspx and https://www.kcl.ac.uk/research/research-environment/rgei/research-ethics/use-of-personal-data-in-research
- by asking one of the research team (contact details included below) or
- by contacting the Data Protection Officer: (For GSTT: Nick Murphy-O'Kane DPO@gstt.nhs.uk; For KCL: Olenka Cogias info-compliance@kcl.ac.uk)

MORE INFORMATION ABOUT TAKING PART

13. What if relevant new information about Mirococept becomes available?

Sometimes during the course of a research project, new information becomes available about what is being studied. To ensure your safety, an independent committee of experts will review the results regularly during the study. They will also look at the results of other relevant studies. They can stop the study early if they see any unfavourable results.

14. What happens if I don't want to carry on with the study?

You are free to withdraw your consent to participate in the study at any time and without giving a reason. This will not affect the standard of care you receive. You have the right to request samples collected as part of this study to be destroyed and no further laboratory analysis to be performed. Data already collected about you will be retained.

Your study doctor can take you out of the study at any time if it is in your best medical interests to stop your participation. The study sponsor also has the right to direct your study doctor to take you out of the study at any time.

If you have any questions that remain unanswered, your study doctor or nurse will be happy to answer them for you.

15. What will happen at the end of the study?

The study is expected to take over two years to complete, starting in 2024. We are hoping to publish the results through medical publications and presentations shortly after completing the study. At this point, we will be happy to send you a summarised version of the study results at your request (your preference will be noted on your Consent Form). You will not be identifiable in the report.

Once your involvement in the study is over, you will continue to receive your usual care.

After completion of the study data collected will be archived for 5 years. These will be stored in a secure location managed by your local hospital Trust.

16. What if there is a problem?

If you have a concern about any aspect of this study, you should ask to speak to the researchers who will do their best to answer your questions [insert Principal Investigator name, telephone number and e-mail address]. If you remain unhappy and

wish to complain formally, you can do this through the [insert local site PALS name, telephone number and e-mail address].

In the event that something does go wrong and you are harmed during the research you may have grounds for legal action for compensation against Guy's and St Thomas' NHS Foundation Trust and/or King's College London but you may have to pay your legal costs. The normal National Health Service complaints mechanisms will still be available to you (if appropriate).

17. Who is organising and funding the study?

The study is funded by the Medical Research Council. The trial is co-sponsored by Guy's and St Thomas' NHS Foundation Trust and King's College London.

18. Who has reviewed the study?

This research has been reviewed by an independent group of people, called a Research Ethics Committee, to protect your safety, rights, wellbeing and dignity. This study has been reviewed and given a favourable opinion by North East - Newcastle & North Tyneside 2 Research Ethics Committee. The study has also been reviewed by the UK Regulatory Authority, the MHRA (the Medicines and Healthcare products Regulatory Agency). The MHRA is part of the Department of Health with the responsibility to regulate clinical trials of medicines in the UK.

Kidney transplant patients were involved in reviewing and providing feedback on this Patient Information Sheet, the Patient Invitation Letter and Informed Consent Form.

Thank you for taking the time to read this Patient Information Sheet.

Contact details: ****PLEASE UPDATE with the site PI, local research team and site PALS details****

Site Principal Investigator details:

Name:

Address:

Email:

Site local research team details:

Name/Team:

Address:

Email:

Tel:

If you have any concerns about the way the study is carried out by the study staff, or your rights as a research patient, or any other aspects of your care, please contact the person below.

Site PALS Team details:

PALS Lead, Patient Information Team

Address:

Email:
