# Supplementary material for: Multicentre, multi-arm, double-blind randomised placebo-controlled dose-finding trial investigating the safety and Efficacy of MirococePt (APT070) In Reducing delayed graft function In the Kidney ALlograft (EMPIRIKAL-2): study protocol for a randomised controlled trial
Source: BMJ Open. 2025 Mar 6;15(3):e097029. doi: 10.1136/bmjopen-2024-097029 (PMC11887295; doi:10.1136/bmjopen-2024-097029)
Supplement: online supplemental file 7 [file bmjopen-15-3-s007.docx]

**Supplemental Table 1: Schedule of Events**

| **Assessment** | **Day 0**  **SC/BL** | | | | **Day 1** | **Day 2** | **Day 3** | **Day 4** | **Day 7** | **Wk 2** | **Wk 3** | **Wk 4** | **Wk 5** | **Wk 6** | **Wk 7** | **Wk 8** | **Wk 9** | **Wk 10** | **Wk 11** | **Wk 12** | **Wk 24** | **Wk 36** | **Wk 52** | **WD** |
| --- | --- | --- | --- | --- | --- | --- | --- | --- | --- | --- | --- | --- | --- | --- | --- | --- | --- | --- | --- | --- | --- | --- | --- | --- |
|  | **Pre-Tx** | **Tx** | **1hr Post-Tx** | **4hrs Post-Tx** |  |  |  |  |  |  |  |  |  |  |  |  |  |  |  |  |  |  |  |  |
| **Visit Window** | **Completed on day** | | | | | | | | **+/- 2 days** | **Anytime in specified week (7-day window)** | | | | | | | | | | | **+/- 2 weeks** | | | **On day** |
| **Informed consent** | x |  |  |  |  |  |  |  |  |  |  |  |  |  |  |  |  |  |  |  |  |  |  |  |
| **Demographics** | X |  |  |  |  |  |  |  |  |  |  |  |  |  |  |  |  |  |  |  |  |  |  |  |
| **Donor Information^1^** | X |  |  |  |  |  |  |  |  |  |  |  |  |  |  |  |  |  |  |  |  |  |  |  |
| **Organ Information^2^** | X |  |  |  |  |  |  |  |  |  |  |  |  |  |  |  |  |  |  |  |  |  |  |  |
| **Medical & Transplant history** | x |  |  |  |  |  |  |  |  |  |  |  |  |  |  |  |  |  |  |  |  |  |  |  |
| **Dialysis (incl. mode)** | x |  |  |  | x | x | x | x | x | x | x | x | x | x | x | x | x | x | x | x | x | x | x | x |
| **Vital signs^3^** | x |  |  |  | x | x | x | x | x | x | x | x | x | x | x | x | x | x | x | x | x | x | x | x |
| **General Physical Examination^4^** | x |  |  |  | Δ | Δ | Δ | Δ | Δ | Δ | Δ | Δ | Δ | Δ | Δ | Δ | Δ | Δ | Δ | Δ | Δ | Δ | Δ | Δ |
| **Serum Pregnancy test^5^** | x |  |  |  |  |  |  |  |  |  |  |  |  |  |  |  |  |  |  |  |  |  |  |  |
| **Virology clinical blood test^6^** | X |  |  |  |  |  |  |  |  |  |  |  |  |  |  |  |  |  |  |  |  |  |  |  |
| **Haematology clinical blood test^7^** | x |  |  |  | x | x | x | x | x | x | x | x | x | x | x | x | x | x | x | x | x | x | x | x |
| **Assessment** | **Day 0**  **SC/BL** | | | | **Day 1** | **Day 2** | **Day 3** | **Day 4** | **Day 7** | **Wk 2** | **Wk 3** | **Wk 4** | **Wk 5** | **Wk 6** | **Wk 7** | **Wk 8** | **Wk 9** | **Wk 10** | **Wk 11** | **Wk 12** | **Wk 24** | **Wk 36** | **Wk 52** | **WD** |
|  | **Pre-Tx** | **Tx** | **1hr Post-Tx** | **4hrs Post-Tx** |  |  |  |  |  |  |  |  |  |  |  |  |  |  |  |  |  |  |  |  |
| **Visit Window** | **Completed on day** | | | | | | | | **+/- 2 days** | **Anytime in specified week (7-day window)** | | | | | | | | | | | **+/- 2 weeks** | | | **On day** |
| **Biochemistry clinical blood test^8^** | x |  |  |  | x | x | x | x | x | x | x | x | x | x | x | x | x | x | x | x | x | x | x | x |
| **Urinalysis^9^** | Δ |  |  |  | Δ | Δ | Δ | Δ | Δ | Δ | Δ | Δ | Δ | Δ | Δ | Δ | Δ | Δ | Δ | Δ | Δ | Δ | Δ | Δ |
| **Inclusion Exclusion criteria check** | x |  |  |  |  |  |  |  |  |  |  |  |  |  |  |  |  |  |  |  |  |  |  |  |
| **Any Adverse Events** | x |  | x | x | x | x | x | x | x | x | x | x | x | x | x | x | x | x | x | x | x | x | x | x |
| **Concomitant medications** | x |  | x | x | x | x | x | x | x | x | x | x | x | x | x | x | x | x | x | x | x | x | x | x |
| **Randomisation** | x |  |  |  |  |  |  |  |  |  |  |  |  |  |  |  |  |  |  |  |  |  |  |  |
| **Tacrolimus trough** |  |  |  |  |  | Δ |  | Δ | Δ | Δ | Δ | Δ | Δ | Δ | Δ | Δ | Δ | Δ | Δ | Δ | Δ | Δ | Δ | Δ |
| **C-Reactive Protein (CRP)** | Δ |  |  |  |  |  |  |  |  |  |  |  |  |  |  |  |  |  |  |  |  |  |  |  |
| **Mirococept (IMP)/placebo perfusion^10^** |  | X |  |  |  |  |  |  |  |  |  |  |  |  |  |  |  |  |  |  |  |  |  |  |
| **Any episode acute rejection^11^** |  |  |  |  | Δ | Δ | Δ | Δ | Δ | Δ | Δ | Δ | Δ | Δ | Δ | Δ | Δ | Δ | Δ | Δ | Δ | Δ | Δ | Δ |
| **Any episode CNI toxicity^11^** |  |  |  |  | Δ | Δ | Δ | Δ | Δ | Δ | Δ | Δ | Δ | Δ | Δ | Δ | Δ | Δ | Δ | Δ | Δ | Δ | Δ | Δ |
| **BK & CMV** |  |  |  |  | Δ | Δ | Δ | Δ | Δ | Δ | Δ | Δ | Δ | Δ | Δ | Δ | Δ | Δ | Δ | Δ | Δ | Δ | Δ | Δ |
| **Assessment** | **Day 0**  **SC/BL** | | | | **Day 1** | **Day 2** | **Day 3** | **Day 4** | **Day 7** | **Wk 2** | **Wk 3** | **Wk 4** | **Wk 5** | **Wk 6** | **Wk 7** | **Wk 8** | **Wk 9** | **Wk 10** | **Wk 11** | **Wk 12** | **Wk 24** | **Wk 36** | **Wk 52** | **WD** |
|  | **Pre-Tx** | **Tx** | **1hr Post-Tx** | **4hrs Post-Tx** |  |  |  |  |  |  |  |  |  |  |  |  |  |  |  |  |  |  |  |  |
| **Visit Window** | **Completed on day** | | | | | | | | **+/- 2 days** | **Anytime in specified week (7-day window)** | | | | | | | | | | | **+/- 2 weeks** | | | **On day** |
| **Urine microbiology** |  |  |  |  |  |  | Δ | Δ | Δ | Δ | Δ | Δ | Δ | Δ | Δ | Δ | Δ | Δ | Δ | Δ | Δ | Δ | Δ | Δ |
| **Development of PTLD** |  |  |  |  |  |  |  |  | Δ | Δ | Δ | Δ | Δ | Δ | Δ | Δ | Δ | Δ | Δ | Δ | Δ | Δ | Δ | Δ |
| **In addition, Research Samples for Guy’s Hospital Participants Only** | | | | | | | | | | | | | | | | | | | | | | | | |
| **Serum Mirococept** | X |  | X^12^ | X^13^ | X | X |  | X |  |  |  |  |  |  |  |  |  |  |  |  |  |  |  |  |
| **Serum antibodies to Mirococept^14^** | X |  |  |  |  |  |  |  |  | X |  | X |  |  |  |  |  | X |  |  |  |  |  |  |
| **Complement (serum CH50%) levels^15^** | X |  | X | X | X | X |  | X^16^ |  |  |  |  |  |  |  |  |  |  |  |  |  |  |  |  |
| **Transplant kidney biopsy^17^ (if consented)** | Δ^18^ |  | Δ^19^ |  |  |  |  |  |  |  |  |  |  |  |  |  |  |  |  |  |  |  |  |  |
| **Blood for biomarkers^20^**  **(if consented)** | Δ |  |  |  | Δ |  |  |  | Δ |  |  | Δ |  |  |  |  |  |  |  |  |  |  |  |  |

1. Donor information to include: Demography (age, sex, race). weight, height, cause of death, premorbid conditions, serum creatinine on admission and pre-donation, creatinine at time of death, blood culture and urine culture (please refer to section 9.1.1)
2. Organ information to include: primary warm ischaemia time, cold ischaemic time and pre-implant biopsy result if performed for clinical indication (please refer to section 9.1.3)
3. Vital signs to include: Blood Pressure (BP), Heart Rate (HR), Respiratory Rate (RR) (desirable), Temperature, Oxygen Saturation (O2 Sats) (desirable), weight and height. Height is only required at Day 0 – pre-transplant timepoint only. Weight is required at Day 0 and desirable thereafter - collected at the discretion of the clinical team
4. No minimum requirements for the physical exam, but at discretion of the assessing investigator and as per routine care.
5. For females of child bearing potential (FOCBP)
6. Virology to include: HIV, HCV and HBV. The last available result from the referring unit will be acceptable.
7. Haematology to include: Haemoglobin, Platelets, Haematocrit, Red Blood Cells (RBC), Mean Cell Volume, White Blood Cell, Lymphocyte, Basophil, Monocyte, Neutrophil and Eosinophils
8. Biochemistry to include: Glucose, Gamma-Glutamyl Transferase (GGT), Potassium, Sodium, Creatinine, total bilirubin, Bicarbonate, Alkaline Phosphatase, Phosphorus, Total Protein, Magnesium, AST/ALT, Albumin, Calcium and eGFR.
9. Urinalysis to include: Protein, Specific Gravity, Glucose, pH, Ketones, leucocytes, nitrites and Urine Sediment.
10. Mirococept (IMP)/Placebo is only administered once to the explanted donor kidney prior to kidney implant (please refer to section 7.2 for administration details).
11. An episode of acute rejection and CNI toxicity are diagnosed from histological analysis of kidney tissue collected via a biopsy clinically indicated as part of the participant’s routine care.
12. 1-hour post reperfusion.
13. 4 hours post reperfusion.
14. Serum antibodies to Mirococept to be collected during the RCT stage only and for participants attending Guy’s Hospital for clinical follow-up only.
15. Complement levels are only needed for the safety run participants and for about10 participants per arm during the RCT stage.
16. Complement levels at Day 4 won’t be required in the randomisation stage if the initial safety run shows it is not needed.
17. Please refer to sections 9.1.6 and 9.1.7.
18. Pre-transplant kidney biopsy should be taken before IMP/placebo perfusion of the donor kidney.
19. Post-transplant kidney biopsy can be taken anytime from reperfusion with blood to before closure of surgical wound.
20. Biomarker samples won’t be collected during the safety run.

| **Key** | | |
| --- | --- | --- |
| **BL** = baseline | **SC** = screening | **WD** = withdrawal |
| **Tx** = transplant | **X** = essential | **Δ** = desirable |
| **Assessment Windows** | | |
| Assessments must be completed on day specified | | |
| Assessments completed **+/- 2 days** of that time point | | |
| Assessments completed **anytime in that 7-day** window | | |
| Assessments completed **+/- 2 weeks** of that time point | | |

The above assessments all form part of the routine management of renal transplant assessments (desirable assessments only required if being collected as part of routine clinical care**)** with the exception of the following research samples taken at Guy’s Hospital only;

- serum for antibodies to Mirococept
- serum for Mirococept levels
- serum of complement activity levels
- renal biopsy specimens
- whole blood for biomarkers

Please refer to the study lab manual for details on collection and processing of the research samples.

**Where an “x” is contained within a field this denotes that the associated data is essential and if missed will be classified as a protocol deviation.**

**Where an “Δ” is contained within a field this denotes that the associated data is desirable and while every effort should be made to record these data if being collected as part of routine clinical care, missing data will NOT constitute a protocol deviation.**
