# Supplementary material for: Multicentre, multi-arm, double-blind randomised placebo-controlled dose-finding trial investigating the safety and Efficacy of MirococePt (APT070) In Reducing delayed graft function In the Kidney ALlograft (EMPIRIKAL-2): study protocol for a randomised controlled trial
Source: BMJ Open. 2025 Mar 6;15(3):e097029. doi: 10.1136/bmjopen-2024-097029 (PMC11887295; doi:10.1136/bmjopen-2024-097029)
Supplement: Supplementary file 8 [file bmjopen-2024-097029.reviewer_comments.pdf]

PEER REVIEW HISTORY

BMJ Open publishes all reviews undertaken for accepted manuscripts. Reviewers are asked to complete a checklist review form and are provided with free text boxes to elaborate on their assessment. These free text comments are reproduced below.

ARTICLE DETAILS

Title (Provisional)

A multi-centre, multi-arm, double-blind randomised placebo-controlled dose finding trial investigating the safety and Efficacy of MirococePt (APT070) In Reducing delayed graft function In the Kidney ALlograft (EMPIRIKAL-2): study protocol for a randomised controlled trial.

Authors

Kassimatis, Theodoros; Rahman, Alima; Jaradat, Fayyad; Douiri, Abdel; Delord, Marc; Greenlaw, Roseanna; Palmer Joyce, Joanne; Olsburgh, Jonathon; Khurram, Muhammad; Ghazanfar, Abbas; Knight, Simon; Friend, Peter; Shah, Sapna; Killbride, Hannah; Smith, Richard; Sacks, Steven

---

VERSION 1 - REVIEW

Reviewer 1

Name Kitamura, Mineaki

Affiliation Nagasaki University School of Medicine Graduate School of Biomedical Sciences, Nephrology

Date 22-Dec-2024

COI None

The authors submitted a protocol paper evaluating the effect of mirococept on delayed graft function in deceased kidney transplant recipients. While the proposed study protocol is well-designed, several concerns need to be addressed.

First, following the EMPRIKAL study, the dosage of mirococept is critical for preventing delayed graft function in kidney transplant recipients. According to Am J Transplant. 2021;21(3):1012-1026, a dose of 80 mg mirococept is considered suitable. However, this study protocol does not account for kidney weight. Instead, it specifies dose ranges between 60–180 mg irrespective of kidney weight. The authors should provide a detailed rationale for this decision and clarify the background for this approach.

Second, machine perfusion has been shown to reduce delayed graft function, as demonstrated in N Engl J Med. 2023;388(5):418-426. However, the study protocol excludes donated kidneys that have undergone machine perfusion. While the study's sample size is

understandably small, it would be beneficial for the authors to consider conducting a stratified analysis of kidneys with and without machine perfusion in future studies.

Third, the authors noted that the recent proportion of delayed graft function at Guy's Hospital is approximately 50%, a rate that has increased due to donor characteristics. It would be helpful for the authors to provide evidence showing that delayed graft function rates are consistent across the facilities participating in this study.

**Reviewer**	**2**
**Name**	**Bentall, Andrew**
**Affiliation**	**Mayo Clinic Rochester, Division of Nephrology and Hypertension**
**Date**	**28-Dec-2024**
**COI**	**No competing interest**

The protocol is an ambitious goal to achieve improved patient outcomes.

I think this is acceptable for publication.

Suggestion, but not an issue is DGF for function vs. DGF for hyperkalemia as a definition - as often post-transplant dialysis is needed for hyperkalemia - which often is not delayed graft function - just increased cellular release of potassium.

VERSION 1 - AUTHOR RESPONSE

Reviewer 1
Dr. Mineaki Kitamura, Nagasaki University School of Medicine Graduate School of Biomedical Sciences
Comments to the Author:
The authors submitted a protocol paper evaluating the effect of mirococept on delayed graft function in deceased kidney transplant recipients. While the proposed study protocol is well-designed, several concerns need to be addressed.

First, following the EMPRIKAL study, the dosage of mirococept is critical for preventing delayed graft function in kidney transplant recipients. According to Am J Transplant. 2021;21(3):1012-1026, a dose of 80 mg mirococept is considered suitable. However, this study protocol does not account for kidney weight. Instead, it specifies dose ranges between 60–180 mg irrespective of kidney weight. The authors should provide a detailed rationale for this decision and clarify the background for this approach.

Thank you for your comment. In our AJT study the optimal dose of Mirococept for a pig kidney (weighing approximately 150g) was 80mg. This dose resulted in

saturation of glomeruli and widespread binding to tubular epithelium. This dose did not induce any additional histological damage compared to controls or after a subsequent 3 hours of normothermic machine perfusion. Scaling up for the average weight of human kidney (225g), the equivalent dose of Mirococept is approximately 120mg (Codas et al. Nephrology (Carlton). 2014;19(7):420-5). The dose of 40mg (human equivalent of 60mg) demonstrated weaker binding in the pig kidney.

Trying to identify a safe upper dose, we went above the 80mg dose defined as optimal in the pig kidney analysis, and used 120mg (human equivalent of 180mg) for a washout study. We estimated that 12.5% of the Mirococept dose was washed out (22.5mg for the human equivalent dose of 180mg), which is well within the systemic dose range considered safe as determined in the Phase I trial.

Second, machine perfusion has been shown to reduce delayed graft function, as demonstrated in N Engl J Med. 2023;388(5):418-426. However, the study protocol excludes donated kidneys that have undergone machine perfusion. While the study's sample size is understandably small, it would be beneficial for the authors to consider conducting a stratified analysis of kidneys with and without machine perfusion in future studies.

Thank you for your comment. Indeed, there is evidence that machine perfusion can reduce the incidence of delayed graft function. Therefore, we have decided not to include kidneys that have undergone machine perfusion (both ex vivo and Normothermic Regional Perfusion (NRP)) as this can affect the primary outcome (delayed graft function). We will keep this under review for future studies.

Third, the authors noted that the recent proportion of delayed graft function at Guy’s Hospital is approximately 50%, a rate that has increased due to donor characteristics. It would be helpful for the authors to provide evidence showing that delayed graft function rates are consistent across the facilities participating in this study.

Thank you for your comment. There are variations in DGF rates among sites because of variable case mix. The base-rate of 50% is consistent with recent Guy’s Hospital audit data and has also been reported in a Cambridge study of 525 consecutive kidney transplants (Transplantation. 2013 Nov 27;96(10):885-9). In the previous EMPIRIKAL study, which was also a multicentre RCT, the DGF rate was 64%, and 71% of recruitment came from Guy’s Hospital. We have therefore powered the study for a DGF base-rate of 50%.

Reviewer: 2
Dr. Andrew Bentall, Mayo Clinic Rochester
Comments to the Author:
The protocol is an ambitious goal to achieve improved patient outcomes.
I think this is acceptable for publication.

Suggestion, but not an issue is DGF for function vs. DGF for hyperkalemia as a definition - as often post-transplant dialysis is needed for hyperkalemia - which often is not delayed graft function - just increased cellular release of potassium.

Thank you for your comment. We have considered this option but decided to proceed with the traditional definition of DGF which was found to be not inferior to any other definition of DGF by Malon et al (Transplantation. 2013 Nov 27;96(10):885-9). The investigators concluded that this definition, which is the most widely used and most easily calculated, should be universally adopted as the definition of DGF clinically and as a study endpoint.

Moreover, given the randomised design of the study, cases with dialysis due to hyperkalaemia should be equally distributed among groups. In any case, we will be recording the indications for haemodialysis including hyperkalaemia, so a subgroup analysis may be possible.
